# scAICT: Adversarial Integration and Contrastive Translation for Unpaired scRNA-Seq and scATAC-Seq

**DOI:** 10.3390/biology15201797

**Published:** 2026-10-09

**Authors:** Xin Wang, Jiale Ren, Qiu Xie, Lin Meng

**Affiliations:** 1 Graduate School of Science and Engineering, Ritsumeikan University, Kusatsu 525-8577, Japan; gr0663vk@ed.ritsumei.ac.jp (X.W.); gr0731xf@ed.ritsumei.ac.jp (J.R.); 2 State Key Laboratory of Complex Severe and Rare Diseases, Peking Union Medical College Hospital, Chinese Academy of Medical Sciences and Peking Union Medical College, Beijing 100730, China; 3 College of Science and Engineering, Ritsumeikan University, Kusatsu 525-8577, Japan

**Keywords:** single-cell multiomics, unpaired data integration, gene expression, chromatin accessibility, cross-modal translation, cell-type annotation, contrastive learning

## Abstract

Unpaired single-cell gene expression and chromatin accessibility data lack the cell-level correspondence needed to directly link measurements across modalities. We present scAICT, a two-stage framework that infers this correspondence through integration and uses it to guide bidirectional molecular prediction. Modality-specific variational autoencoders combine adversarial alignment with structural and anchor constraints to learn a shared representation. Inferred pseudo-pairs then supervise directional mapping networks through cross-modal reconstruction and contrastive matching. We evaluate scAICT on naturally unpaired Muto-2021 kidney data and PBMC-10k data under unpaired training, with a complementary paired PBMC-10k translation benchmark. scAICT achieves the highest overall integration scores among the evaluated methods, reaching 78.62% on Muto-2021 and 84.61% on PBMC-10k, and supports cross-modal annotation transfer. Under unpaired training, its mean biological conservation scores exceed Monae in both directions, with larger gains for gene expression prediction from chromatin accessibility. PBMC-10k molecular evaluations also show higher median cell-level correlations than Monae in both directions, including in cells held out from translation training. Predicted RNA retains cell-type-associated marker patterns. These findings support molecular prediction guided by inferred correspondence, with evidence for cell-type conservation in kidney data and molecular-profile concordance in PBMC-10k.

## Simple Summary

Measurements of gene expression and chromatin accessibility provide complementary information about cell identity and gene regulation. Although current technologies can collect both measurements from the same cell, many datasets measure them in separate cells, leaving no direct match between the two types of information. We develop a computational method, scAICT, that first links cells with similar biological characteristics across the two measurements and then uses these inferred links to predict one measurement from the other. Analyses of human kidney and blood data show that the method preserves distinctions among cell types and supports the transfer of cell labels between measurements. In blood data, predictions agree with measured molecular profiles and retain recognizable immune-cell expression patterns even when true cell matches are unavailable during training. These findings support the use of inferred cell relationships to predict missing molecular profiles; validation in kidney data remains limited to preservation of cell-type structure.

## 1. Introduction

Understanding how gene regulation shapes cellular identity and state is a fundamental goal of single-cell biology [1]. Single-cell RNA sequencing (scRNA-seq) measures gene expression, whereas single-cell assay for transposase-accessible chromatin sequencing (scATAC-seq) profiles accessible genomic regions [2,3,4]. These complementary measurements connect transcriptional patterns with their associated chromatin landscapes, providing a basis for investigating gene regulation across cellular identities and states [5,6,7,8,9]. In practice, RNA and ATAC profiles often originate from independently measured cells, leaving no direct cell-level correspondence between the modalities [10,11,12]. Although joint profiling technologies provide matched measurements, they do not supply the missing counterparts for existing independently generated datasets [6,9]. Computational analysis of unpaired data therefore requires correspondence to be inferred from the information shared across modalities [5,13]. Establishing this correspondence can support the transfer of cell-type annotations and the prediction of molecular profiles that are unavailable for individual cells [14,15,16].

Integrating unpaired gene expression and chromatin accessibility data is challenging because the modalities differ in both the cells measured and the features observed [10]. Existing methods infer correspondence by drawing on feature relationships and the organization of cells within each modality. Feature-based approaches use accessibility-derived gene activity or learned feature associations to connect the two measurement spaces [17,18]. These connections support cell matching, as illustrated by anchor identification in Seurat_v3 and iterative matching with weakly linked features in MaxFuse [19,20]. Regulatory information further informs correspondence through feature guidance graphs in scGLUE and the joint modeling of cellular heterogeneity and transcriptional regulatory links in FGOT [5,10]. Complementary approaches exploit within-modality cellular relationships through manifold geometry, optimal transport, or graph representations [21,22,23]. Adversarial and optimal-transport frameworks incorporate constraints on cellular structure or cross-modal relationships to guide distributional alignment [11,24,25]. Overall, these strategies infer cross-modal relationships from independently measured cells using different sources of shared information. However, overlapping modality distributions alone may not be sufficient to establish correct cell-type correspondence [12,24]. Integrating unpaired gene expression and chromatin accessibility data therefore requires inferring cross-modal correspondence in the absence of known cell-level pairings while preserving biologically meaningful differences among cell types.

Cross-modal translation aims to predict an unobserved molecular profile from measurements of another modality, extending single-cell analysis to molecular information unavailable for individual cells [26]. Many existing approaches use paired measurements or available cross-modal matches to learn the relationships needed for prediction. BABEL learns interoperable encoders and decoders from jointly measured gene expression and chromatin accessibility profiles, whereas JAMIE incorporates available matches into joint embedding and imputation [27,28]. Paired reference data can also connect independently measured datasets. For example, scPairing learns a common representation from a multiomics bridge and constructs artificial multiomics datasets by pairing unimodal profiles [29]. Across these approaches, observed correspondence provides either direct training targets or a reference for linking modalities. For unpaired data without a paired reference, the relationships needed for prediction must instead be inferred from the available molecular profiles. scButterfly learns cross-modal translation from paired measurements and also provides an unpaired variant that constructs training pairs using known cell-type annotations from both modalities [30]. Monae learns shared cell representations from unpaired data and combines them with graph-linked target-feature embeddings to generate cross-modal predictions without requiring native cell correspondences [15].

Here, we present scAICT, a framework that connects integration and bidirectional translation of unpaired single-cell gene expression and chromatin accessibility data. During integration, modality-specific variational autoencoders learn cellular representations, while adversarial alignment reduces differences between their latent distributions. Within-modality structural constraints preserve cellular relationships, and cross-modal anchors guide alignment between biologically related cells. The resulting shared representation supports annotation transfer and provides inferred pseudo-pairs for translation training. In the translation stage, two directional mapping networks transform source representations into the target latent space, where modality-specific decoders generate predicted molecular profiles. Cross-modal reconstruction and contrastive matching use the inferred pairs to guide prediction, while self-reconstruction preserves modality-specific information and mapped-latent regularization constrains the translated representations. By assigning distinct objectives to the two stages, scAICT uses correspondence learned during integration to guide molecular prediction without requiring native cell-level pairing.

We evaluate scAICT on the naturally unpaired human kidney dataset Muto-2021 [31] and the paired PBMC-10k dataset [32]. Native PBMC-10k correspondences are withheld during unpaired training and are available during translation training in a complementary paired benchmark. Across both datasets, scAICT achieves the highest overall integration score among the evaluated methods and supports cross-modal annotation transfer. Under unpaired training, translated representations retain cell-type structure more strongly than Monae on average in both directions. PBMC-10k evaluations extend this comparison to decoded molecular profiles, including predictions for cells excluded from translation training, with larger advantages for RNA prediction. An analysis of inferred pseudo-pairs characterizes their ability to recover cell identity and cell type. Together, these evaluations connect correspondence inference with structural preservation and molecular prediction, while the absence of matched kidney measurements limits molecular validation to PBMC-10k.

## 2. Materials and Methods

### 2.1. Overview of scAICT

**Figure 1:**
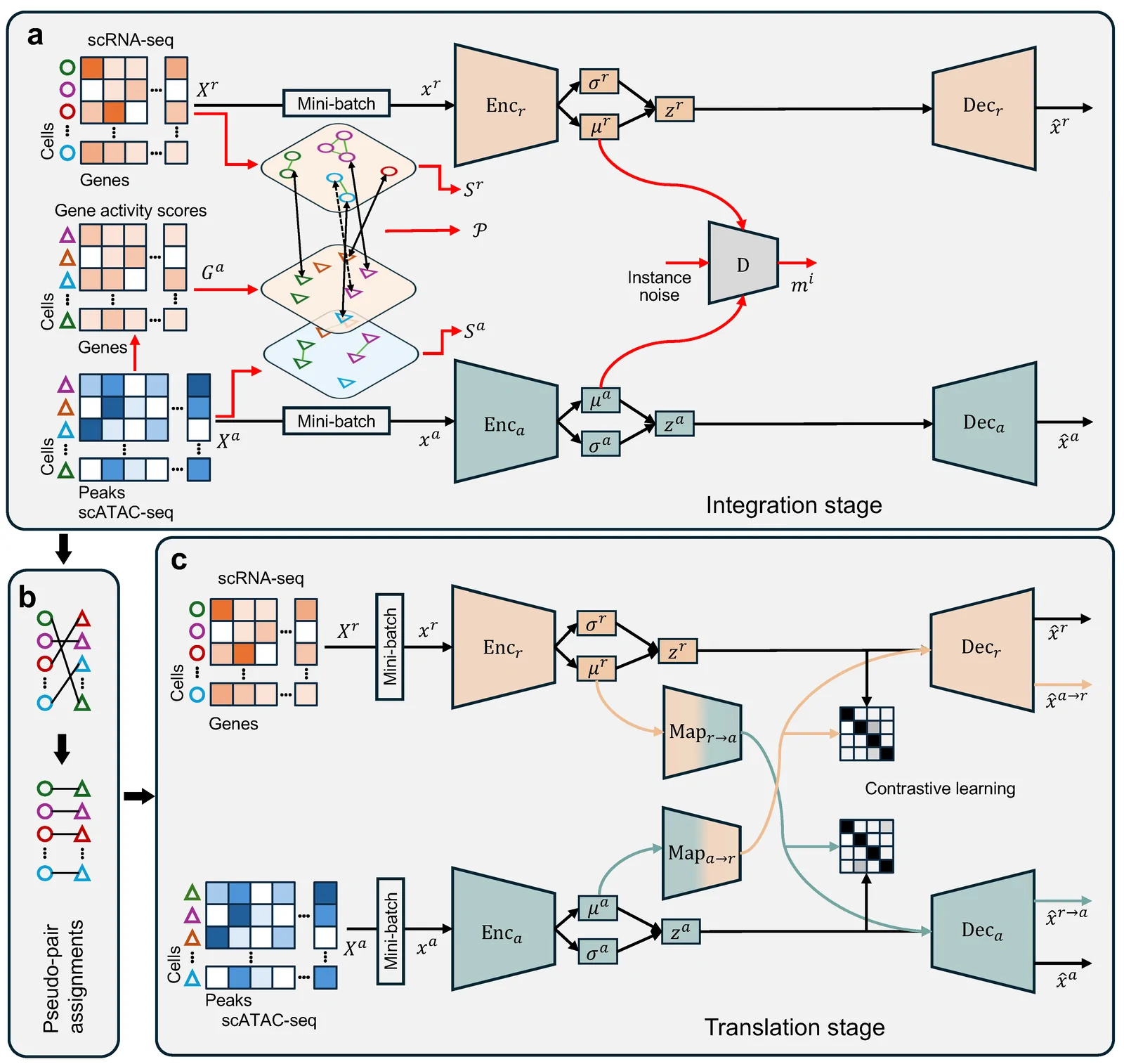
Two-stage workflow of scAICT for unpaired integration and bidirectional translation. (**a**) modality-specific encoders and decoders learn latent representations and reconstruct RNA and ATAC profiles. Adversarial training with discriminator *D* (output $m^{i}$; instance noise is added to its inputs) promotes modality alignment. Structural constraints based on $S^{r}$ and $S^{a}$ preserve within-modality relationships, while the cross-modal anchor set P guides alignment of cellular representations. ATAC-derived gene activity scores $G^{a}$ support anchor construction. (**b**) nearest-neighbor matching in the integrated latent space supplies fixed pseudo-pair assignments for translation. (**c**) independently initialized autoencoders and directional mapping networks learn RNA-to-ATAC and ATAC-to-RNA prediction using these assignments. The matrices illustrate contrastive matching between mapped representations and their assigned targets. Superscripts *r* and *a* denote RNA and ATAC, respectively; μ, σ, and *z* denote posterior means, standard deviations, and sampled latent representations. Cell colors schematically indicate biological populations; pseudo-pairs need not recover exact cell identity or always match cell type. Circles and triangles denote RNA and ATAC cells; orange and teal denote RNA- and ATAC-related components, data, and arrows. Black arrows denote data flow, and red arrows in (**a**) indicate inputs to the alignment terms. Ellipses denote omitted rows and columns.

scAICT connects integration and bidirectional translation of single-cell RNA and ATAC profiles through two stages (Figure 1). During integration, modality-specific variational autoencoders learn cellular representations while reconstructing the observed counts. Adversarial alignment reduces modality differences, while structural and anchor constraints preserve within-modality relationships and guide cross-modal correspondence. The integrated representations support independent integration analyses and provide nearest-neighbor pseudo-pairs for unpaired translation. During translation, two directional mapping networks transform source posterior means into the target latent space, where the corresponding decoders generate predicted profiles. Pseudo-pairs guide cross-modal reconstruction and contrastive matching, while self-reconstruction retains modality-specific information. In this study, the translation-stage autoencoders and mapping networks are initialized independently, with integration supplying only the pseudo-pair assignments.

#### 2.1.1. Modality-Specific Representation Learning

RNA expression and chromatin accessibility describe different aspects of cellular states and have distinct feature spaces. To capture their respective information, scAICT employs two modality-specific variational autoencoders [33]. Let $X^{r}$ and $X^{a}$ denote the RNA and ATAC count matrices, respectively. Each encoder normalizes the input counts to a total of $10^{4}$ per cell, applies a log(1+x) transformation, and extracts a low-dimensional representation through two fully connected hidden layers. The encoding process is expressed as

(1) $$\left(\mu^{v},\sigma^{v}\right)={\mathrm{Enc}}_{v}\left(X^{v}\right),\;Z^{v}=\mu^{v}+\sigma^{v}⊙E^{v},\;v\in \left\{r,a\right\},$$

where $\mu^{v}$ and $\sigma^{v}$ are the posterior means and positive standard deviations, respectively, and the entries of $E^{v}$ follow a standard Gaussian distribution. A softplus transformation ensures that the estimated standard deviations are positive. The *i*-th row of $Z^{v}$, denoted by $z_{i}^{v}$, represents the sampled latent vector of cell *i*. Both modalities use a latent dimension of d=32.

The decoder reconstructs the observed counts from the latent representation using a negative binomial likelihood, a count-modeling approach used in single-cell generative models [34]. Specifically, the reconstructed mean is obtained as ${\hat{x}}_{i}^{v}=\ell_{i}^{v}\mathrm{softmax}\left(f_{\theta_{v}}\left(z_{i}^{v}\right)\right)$, where $f_{\theta_{v}}$ produces the decoder logits and $\ell_{i}^{v}$ is the total count over the selected features. The reconstruction loss for modality *v* is defined as

(2) $$L_{\mathrm{rec}}^{v}=-\frac{1}{B_{v}D_{v}}\sum_{i=1}^{B_{v}}\sum_{j=1}^{D_{v}}\log\mathrm{NB}\left(x_{ij}^{v};{\hat{x}}_{ij}^{v},\vartheta_{b_{i}j}^{v}\right),$$

where $B_{v}$ is the number of cells in the training mini-batch, $D_{v}$ is the number of input features, and NB denotes the negative binomial distribution parameterized by its mean and dispersion. The index $b_{i}$ identifies the experimental batch of cell *i* in the sample metadata and is independent of training mini-batch membership. The positive dispersion parameter $\vartheta_{b_{i}j}^{v}$ is learned for each feature and experimental batch, with a single feature-specific dispersion vector used when batch information is not supplied.

To regularize the latent representations, the approximate posterior is constrained toward a standard Gaussian prior. For the diagonal Gaussian posterior specified by the encoder outputs, the KL divergence has the following closed form:

(3) $$L_{\mathrm{KL}}^{v}=\frac{1}{2B_{v}D_{v}}\sum_{i=1}^{B_{v}}\sum_{k=1}^{d}\left[{\left(\mu_{ik}^{v}\right)}^{2}+{\left(\sigma_{ik}^{v}\right)}^{2}-\log\left({\left(\sigma_{ik}^{v}\right)}^{2}\right)-1\right],$$

where $\mu_{ik}^{v}$ and $\sigma_{ik}^{v}$ denote the posterior mean and standard deviation of the *k*-th latent component, respectively. The divergence is summed over the *d* latent dimensions and averaged across cells. We additionally divide by $D_{v}$ to apply the same feature-count scaling as in the reconstruction loss (Equation (2)). This common factor scales each modality-specific objective without changing the relative weighting of its reconstruction and KL terms. It reduces the influence of unequal RNA and ATAC feature counts on the combined objective, rather than averaging the KL divergence over latent dimensions.

Combining reconstruction and posterior regularization gives the representation learning objective:

(4) $$L_{\mathrm{rep}}=\sum_{v\in \{r,a\}}\left(\lambda_{\mathrm{rec}}L_{\mathrm{rec}}^{v}+\lambda_{\mathrm{KL}}L_{\mathrm{KL}}^{v}\right),$$

where $\lambda_{\mathrm{rec}}$ and $\lambda_{\mathrm{KL}}$ control the contributions of reconstruction and posterior regularization, respectively.

#### 2.1.2. Adversarial Integration with Structural and Anchor Constraints

The modality-specific autoencoders retain information from their respective inputs, but do not directly establish correspondence between RNA and ATAC profiles. To align their representations, a discriminator predicts the modality label from each cell’s posterior mean. Gaussian instance noise is added before classification, with its standard deviation set to one-half of the corresponding latent dimension’s standard deviation in the combined mini-batch. Experimental batch indicators are also supplied when available. The discriminator minimizes the modality classification cross-entropy, denoted by $L_{\mathrm{adv}}$, while the encoders receive the opposite objective. This adversarial interaction encourages representations that are less distinguishable by modality, following the principle of domain-adversarial representation learning [35].

To preserve cellular relationships during alignment, scAICT further introduces a within-modality structural constraint. For each modality, normalized and log-transformed counts are projected onto 50 principal components to obtain a fixed reference representation $S^{v}$. Within a mini-batch, pairwise Gaussian affinities are calculated separately from the reference representation and the current posterior means. For a representation matrix *R* with row vectors $r_{i}$, the affinity is defined as

(5) $$A{\left(R\right)}_{ij}=\exp\left(-\frac{∥r_{i}-r_{j}{∥}_{2}^{2}}{2h_{R}^{2}}\right),$$

where $h_{R}$ is the median positive pairwise distance in representation *R*, bounded below by a small positive constant. A value of one is used when no positive pairwise distance exists. The structural constraint is defined as

(6) $$L_{\mathrm{stru}}^{v}=\mathrm{MSE}\left(A\left(\mu^{v}\right),A\left(S^{v}\right)\right),\;L_{\mathrm{stru}}=\sum_{v\in \{r,a\}}L_{\mathrm{stru}}^{v},$$

where $\mu^{v}$ and $S^{v}$ contain the posterior means and reference representations of the same cells in the current mini-batch, respectively. The operator MSE denotes the squared difference averaged over all corresponding matrix entries. Separate bandwidths are calculated for the two representations, allowing the constraint to compare relative cell relationships despite differences in their dimensions and scales.

The structural constraint preserves relationships within each modality. To connect the two modalities, cross-modal anchors are constructed using RNA expression and ATAC-derived gene activity. Let *H* denote the binary gene–peak association matrix, where an entry equals one if a peak overlaps the corresponding gene body or its upstream extension. The gene-level ATAC representation is obtained as

(7) $$G^{a}=X^{a}H^{T},$$

where $X^{a}$ contains the peak counts used for anchor construction. Each entry of $G^{a}$ therefore aggregates the accessibility counts associated with a selected gene. By default, the processed peak matrix is used; the implementation also supports a supplied pre-filtering peak matrix restricted to the retained ATAC cells. RNA expression and ATAC gene activity are each normalized to a total of $10^{4}$ per cell and log-transformed, providing a shared gene-level feature space.

To improve the reliability of the gene-level features, neighborhood information is incorporated before cross-modal matching, as in neighborhood-smoothing strategies for weakly linked features [20]. Within each modality, a neighbor graph is constructed from a 50-dimensional PCA representation of the library-size-normalized and log-transformed feature matrix, using gene expression for RNA and peak accessibility for ATAC. Each gene-level feature vector is then combined with the mean vector of its 15 nearest neighbors, excluding the cell itself, with a weight of 0.5 assigned to each component. After $\ell_{2}$ normalization, cross-modal anchors are identified through mutual-nearest-neighbor matching [19] based on cosine similarity, with 30 neighbors considered in each direction across the full dataset.

Let P denote the global anchor set and $P_{B}$ denote the anchor pairs sampled for an optimization step. The anchor constraint is defined as

(8) $$L_{\mathrm{anc}}=\mathrm{MSE}\left(\mu_{P_{B,r}}^{r},\mu_{P_{B,a}}^{a}\right),$$

where $P_{B,r}$ and $P_{B,a}$ index the RNA and ATAC members of the sampled anchor pairs, respectively. The loss is evaluated on their unnormalized posterior means, with anchor pairs sampled independently of the reconstruction mini-batches. Anchor construction does not use cell-type labels. When anchor refreshing is enabled, the global anchor set is periodically recomputed through mutual-nearest-neighbor matching using the current $\ell_{2}$-normalized posterior means.

The autoencoders are optimized for cross-modal integration using the following objective:

(9) $$L_{\mathrm{int}}=L_{\mathrm{rep}}-\lambda_{\mathrm{adv}}L_{\mathrm{adv}}+\lambda_{\mathrm{stru}}\left(t\right)L_{\mathrm{stru}}+\lambda_{\mathrm{anc}}\left(t\right)L_{\mathrm{anc}},$$

where *t* denotes the training epoch. The structural and anchor weights decrease linearly to their respective lower bounds and remain fixed thereafter, while the adversarial weight remains constant. The discriminator separately minimizes $\lambda_{\mathrm{adv}}L_{\mathrm{adv}}$, and its updates alternate with the autoencoder updates. The combined objective aligns the modalities while maintaining within-modality organization and cross-modal correspondence.

#### 2.1.3. Contrastive Bidirectional Translation

In addition to integration, scAICT supports bidirectional prediction between RNA expression and chromatin accessibility. Two directional mapping networks transform the source representations, which are subsequently decoded into target profiles:

(10) $${\hat{X}}^{r\to a}={\mathrm{Dec}}_{a}\left({\mathrm{Map}}_{r\to a}\left(\mu^{r}\right)\right),\;{\hat{X}}^{a\to r}={\mathrm{Dec}}_{r}\left({\mathrm{Map}}_{a\to r}\left(\mu^{a}\right)\right).$$

The decoder expressions omit the depth and experimental batch inputs for simplicity. Each mapping network contains three input-conditioned affine transformation layers. A gating network assigns softmax weights to learnable affine matrices and offsets, allowing the transformation to vary with the input cell. These weights are shared across the three transformation layers within each mapping network, with separate affine parameters for each layer. Parametric rectified linear unit (PReLU) activations [36] are applied between successive layers.

Translation is guided by observed or inferred correspondence between modalities. Available training pairs provide directly matched profiles. For unpaired data, correspondence is inferred from a previously trained integration model. Each RNA profile is assigned its nearest ATAC neighbor in the $\ell_{2}$-normalized latent space, yielding fixed pseudo-pairs for translation training. The same pair assignments support both translation directions. The translation-stage autoencoders can either inherit the integration-stage parameters or be initialized independently. In this study, the autoencoders and directional mapping networks are initialized afresh for translation training; integration supplies the inferred pseudo-pair assignments rather than pretrained model parameters. These translation targets are distinct from the mutual-nearest-neighbor anchors used to optimize integration.

Cross-modal reconstruction is evaluated using the negative binomial likelihood of the assigned target profile. The assigned target profile’s depth is used for both native training pairs and fixed nearest-neighbor pseudo-pairs. For each direction, the negative log-likelihood is averaged over the training pairs and target features. The two directional losses are then summed to obtain $L_{\mathrm{cross}}$.

To encourage translated representations to retain their target correspondence, an InfoNCE-style contrastive objective [37] is applied before decoding. Let *U* contain mapped representations and *V* contain the posterior means of their assigned targets. Their contrastive matching loss is defined as

(11) $$C\left(U,V\right)=-\frac{1}{B}\sum_{i=1}^{B}\log\frac{\exp\left(\cos(u_{i},v_{i})/\tau \right)}{\sum_{j=1}^{B}\exp\left(\cos(u_{i},v_{j})/\tau \right)},$$

where *B* is the number of correspondence pairs in a training mini-batch, cos(·,·) denotes cosine similarity, and τ is the temperature parameter. The diagonal similarity entries correspond to assigned positive pairs, while the off-diagonal entries are treated as negatives without masking repeated targets or cells of the same type. Applying this objective in both directions gives

(12) $$L_{\mathrm{con}}^{r\to a}=C\left({\mathrm{Map}}_{r\to a}\left(\mu^{r}\right),\mu^{a}\right),\;L_{\mathrm{con}}^{a\to r}=C\left({\mathrm{Map}}_{a\to r}\left(\mu^{a}\right),\mu^{r}\right),$$

and

(13) $$L_{\mathrm{con}}=L_{\mathrm{con}}^{r\to a}+L_{\mathrm{con}}^{a\to r}.$$

The target representations in these expressions are ordered according to the training pair assignments.

Mapped representations are further regularized toward the standard Gaussian prior. Specifically, each mapped vector is treated as the mean of a Gaussian distribution with identity covariance. Its KL divergence from the prior reduces to one-half of its squared norm. Averaging over cells and latent dimensions for each direction and summing the two terms gives the mapped-latent regularizer $L_{\mathrm{map}}$. Meanwhile, self-reconstruction and posterior KL regularization are retained to preserve modality-specific information. Self-reconstruction during translation training uses posterior means instead of sampled latent vectors.

The complete translation objective is

(14) $$L_{\mathrm{trans}}=L_{\mathrm{self}}+\lambda_{\mathrm{cross}}L_{\mathrm{cross}}+\lambda_{\mathrm{con}}L_{\mathrm{con}}+\lambda_{\mathrm{map}}L_{\mathrm{map}},$$

where $L_{\mathrm{self}}$ has the same weighted reconstruction and posterior KL terms as $L_{\mathrm{rep}}$ in Equation (4), except that reconstruction is evaluated using the posterior means rather than reparameterized latent samples. Both mapping networks and the modality-specific autoencoders are jointly updated during translation training. The adversarial, structural, and anchor objectives used for integration are not applied during translation training.

### 2.2. Datasets and Preprocessing

We use Muto-2021 human kidney data and PBMC-10k blood data to evaluate integration and translation in naturally unpaired and paired datasets, respectively. For both datasets, we use the preprocessed RNA and ATAC files distributed through the scGLUE data repository [10] as inputs to the preprocessing described below. Muto-2021 contains single-nucleus RNA-seq and ATAC-seq profiles from the adult human kidney [31], comprising 19,985 RNA profiles and 24,205 ATAC profiles spanning 13 annotated cell types. The two modalities are measured separately and do not provide matched observations from individual nuclei. This dataset supports evaluation of integration, annotation transfer, and cell-type conservation during unpaired bidirectional translation.

PBMC-10k contains paired gene expression and chromatin accessibility profiles generated using the 10x Genomics Multiome platform [32]. It comprises 9631 cells spanning 19 annotated cell types, including T cells, B cells, monocytes, and natural killer cells. Native cell correspondences are withheld during integration and simulated unpaired translation and are used only as evaluation references in these settings. In the separate paired translation benchmark, native correspondences are available for translation training. PBMC-10k also supports annotation-transfer evaluation and a separate benchmark of five translation methods in the paired setting. The cell-type annotations supplied with both datasets are retained. Table 1 summarizes the numbers of profiles, genes, peaks, and cell types.

A Scanpy-based pipeline is used to preprocess the RNA and ATAC data. For RNA data, cells with fewer than 200 detected genes or more than 20% mitochondrial counts are removed, and genes detected in fewer than three cells are excluded. Mitochondrial genes are identified by the MT- prefix after converting gene names to uppercase. For ATAC data, cells with fewer than 1000 total peak counts are removed, and peaks detected in fewer than 10 cells are excluded. Quality control is performed independently for each modality. For Muto-2021, the two cell populations are retained independently because individual nuclei are not paired across modalities. For PBMC-10k, shared cell identifiers are subsequently used to retain matched RNA and ATAC profiles.

After quality control, we select the top 3000 highly variable genes for each dataset using the Seurat v3 procedure implemented in Scanpy, with gene variability estimated from the RNA count matrix. Gene and peak coordinates are obtained from the GRCh38/hg38 annotations supplied with the input data. To restrict the ATAC feature space to regions associated with the selected genes, each gene interval is extended by 2000 bp upstream according to its strand. Peaks overlapping at least one resulting interval are retained using pybedtools (v0.12.0) and BEDTools (v2.31.1). Thus, the retained peaks cover selected gene bodies and their upstream regions. This procedure yields 18,229 ATAC peaks for Muto-2021 and 17,873 ATAC peaks for PBMC-10k.

The count matrices are preserved for the selected RNA and ATAC features. In addition, RNA expression is normalized to a total of 10,000 counts per cell and transformed using log(1+x), while ATAC accessibility is binarized by assigning a value of one to positive counts and zero otherwise. These transformed representations are stored alongside the preserved counts. The preserved count matrices serve as inputs to scAICT; model-specific normalization, gene activity calculation, and construction of structural references are described in Section 2.1.

### 2.3. Competing Methods

#### 2.3.1. Integration Methods

We compare scAICT with five methods representing anchor-based, iterative matching, optimal-transport, and deep representation-learning strategies. Seurat_v3 [19] uses canonical correlation analysis to construct a shared subspace and identifies mutual-nearest-neighbor anchors for data alignment and label transfer. MaxFuse [20] iteratively refines cell matching through fuzzy smoothing and co-embedding, allowing integration when cross-modal feature links are weak or noisy. scMODAL [25] uses a limited set of known positively correlated features to identify cross-modal cell anchors. These anchors guide adversarial alignment, while a topology-preserving constraint retains cellular relationships derived from the full input feature spaces. scCobra [38] employs a contrastive variational autoencoder, domain-specific batch normalization, and a domain discriminator to learn domain-invariant representations. For RNA and ATAC integration, it first converts ATAC profiles into gene-activity matrices. scConfluence [11] combines modality-specific autoencoders with regularized inverse optimal transport, using weakly connected features to align unpaired modalities while retaining their complete feature spaces.

#### 2.3.2. Translation Methods

We organize translation comparisons by the supervision available during training. scButterfly learns from paired measurements, while its released unpaired variant uses known cell-type annotations from both modalities to construct training pairs [30]. This introduces label-based supervision absent from our unpaired translation setting. We therefore select Monae [15] as the sole baseline for this comparison because it supports cross-modal prediction without native cell matches or cell-type labels. It learns shared cell representations through graph-encoded regulatory links and contrastive learning, and combines these representations with target-modality feature embeddings to generate predictions without native cell matches. This provides a comparison between prediction from a shared representation and the pseudo-pair-supervised directional mapping used by scAICT. Monae is evaluated exclusively in the unpaired benchmark.

The paired PBMC-10k benchmark compares scAICT with four methods. scButterfly [30] combines dual-aligned variational autoencoders, a latent translator, and adversarial discriminators to predict profiles between modalities. We evaluate it here in the paired setting. UnitedNet [39] is an explainable multi-task network that jointly performs multimodal integration, cross-modal prediction, and cell-type classification within an end-to-end architecture. MultiVI [40] uses a shared probabilistic latent space with modality-specific likelihoods to integrate paired and unpaired RNA and ATAC data and impute missing modalities. JAMIE [28] uses joint variational autoencoders and complete or partial cell correspondences to aggregate modality-specific embeddings, enabling multimodal integration and cross-modal imputation.

### 2.4. Evaluation Metrics

Integration and translation are evaluated using task-specific criteria. Integration quality is measured by biological conservation and modality alignment in the joint embedding of RNA and ATAC profiles. Metric selection follows the single-cell integration benchmarking (scIB) framework [41], with batch-mixing criteria adapted to RNA and ATAC modality labels. The specific metric implementations and aggregation rules used in this study are defined below. Cross-modal label transfer provides an additional evaluation of the integrated representation through cell-type annotation. Translation assessment examines cell-type structure in the direction-specific mapped embeddings and agreement between predicted and observed molecular profiles. All embedding-based metrics are calculated before projection with Uniform Manifold Approximation and Projection (UMAP) [42]. Higher values indicate better performance for all reported scores.

#### 2.4.1. Biological Conservation Metrics

Biological conservation is evaluated using normalized mutual information (NMI), adjusted Rand index (ARI), cell-type average silhouette width (cASW), and cell-type local inverse Simpson’s index (cLISI). Let *N* denote the number of evaluated cells, *C* the number of annotated cell types, and $I_{c}$ the set of cells of type *c*.

NMI measures agreement between cell-type annotations *Y* and inferred cluster assignments *K*:

(15) $$S_{\mathrm{NMI}}=\frac{2I(Y;K)}{H(Y)+H(K)},$$

where I(Y;K) is their mutual information and H(Y) and H(K) are their Shannon entropies, calculated from empirical label frequencies.

ARI measures cell-pair agreement after correction for chance:

(16) SARI=T−AB/N2(A+B)/2−AB/N2,

where T=∑c,knck2, A=∑cnc·2, and B=∑kn·k2. Here, $n_{ck}$ is the number of cells belonging to annotated type *c* and inferred cluster *k*, while $n_{c\cdot }$ and $n_{\cdot k}$ are the corresponding marginal counts. The binomial coefficient n2 counts unordered cell pairs. For NMI and ARI, Leiden clustering is performed on a Scanpy nearest-neighbor graph at resolutions from 0.1 to 2.0 in increments of 0.1, using two iterations per resolution. The maximum NMI and maximum ARI are retained independently.

cASW measures cell-type separation using Euclidean distances:

(17) $$s_{i}=\frac{b_{i}-a_{i}}{\max(a_{i},b_{i})},\;S_{\mathrm{cASW}}=\frac{1}{2}\left[1+\frac{1}{C}\sum_{c=1}^{C}\frac{1}{|I_{c}|}\sum_{i\in I_{c}}s_{i}\right],\;$$

where $a_{i}$ is the mean distance from cell *i* to other cells of the same type, and $b_{i}$ is the minimum mean distance to cells of another type. Singleton-cell silhouettes are assigned zero. Averaging first within cell types and then across types gives equal weight to each type.

cLISI measures local cell-type purity:

(18) $$L_{i}^{\mathrm{cell}}={\left(\sum_{c=1}^{C}p_{ic}^{2}\right)}^{-1},\;S_{\mathrm{cLISI}}=\frac{C-{\bar{L}}^{\mathrm{cell}}}{C-1},$$

where $p_{ic}$ is the fraction of cell *i*’s 30 nearest neighbors assigned to type *c*. Neighbors are identified using Euclidean distance, excluding the cell itself, and are equally weighted. The mean ${\bar{L}}^{\mathrm{cell}}$ is calculated first within cell types and then across types. A score of one is assigned when C=1.

For integration, the four biological conservation metrics are calculated on the combined RNA and ATAC posterior means, using their respective cell-type annotations. The integration biological conservation score is defined as

(19) $$S_{\mathrm{bio}}^{\mathrm{int}}=\frac{1}{4}\sum_{q\in B}S_{q}^{\mathrm{int}},\;B=\left\{\mathrm{NMI},\mathrm{ARI},\mathrm{cASW},\mathrm{cLISI}\right\},\;$$

where B denotes the set of biological conservation metrics and the superscript int indicates evaluation on the combined embedding.

#### 2.4.2. Integration Performance

Modality alignment is assessed using a neighborhood mixing acceptance score reported as kBET, graph connectivity (GC), modality average silhouette width (oASW), and modality local inverse Simpson’s index (iLISI). Modality labels distinguish RNA from ATAC and are separate from experimental batch annotations.

For the reported kBET implementation, observed modality counts among each cell’s 50 nearest neighbors are compared with a balanced reference of 25 RNA and 25 ATAC cells. The cell itself is excluded from its neighborhood. The observed and reference counts form the rows of a 2×2 contingency table, which is evaluated using a chi-square test with continuity correction. The acceptance score is

(20) $$S_{\mathrm{kBET}}=\frac{1}{N}\sum_{i=1}^{N}1\left(p_{i}>0.05\right),\;$$

where $p_{i}$ is the test *p*-value for cell *i* and 1(·) is the indicator function. This implementation evaluates local agreement with balanced modality composition.

GC measures whether cells of the same type remain connected:

(21) $$S_{\mathrm{GC}}=\frac{1}{C}\sum_{c=1}^{C}\frac{|K_{c}|}{|I_{c}|},$$

where $K_{c}$ is the largest connected component after restricting the Scanpy neighbor connectivity graph to cells of type *c*.

oASW measures modality mixing within cell types:

(22) $$S_{\mathrm{oASW}}=\frac{1}{C}\sum_{c=1}^{C}\frac{1}{|I_{c}|}\sum_{i\in I_{c}}\left(1-|s_{i}^{\mathrm{mod}}|\right),$$

where $s_{i}^{\mathrm{mod}}$ is the silhouette value calculated using modality labels within cell *i*’s annotated type. Undefined modality silhouette values are assigned zero before this transformation.

iLISI measures local modality diversity:

(23) $$L_{i}^{\mathrm{mod}}={\left(\sum_{m=1}^{M}\pi_{im}^{2}\right)}^{-1},\;S_{\mathrm{iLISI}}=\frac{{\bar{L}}^{\mathrm{mod}}-1}{M-1},$$

where $\pi_{im}$ is the fraction of cell *i*’s 30 nearest neighbors belonging to modality *m*, and M=2 is the number of modalities. Neighborhoods are constructed as for cLISI. The mean ${\bar{L}}^{\mathrm{mod}}$ is calculated first within modalities and then across modalities.

The modality alignment and overall integration scores are defined as

(24) $$\begin{array}{cc} S_{\mathrm{align}} & =\frac{S_{\mathrm{kBET}}+S_{\mathrm{GC}}+S_{\mathrm{oASW}}+S_{\mathrm{iLISI}}}{4},\\ S_{\mathrm{overall}} & =\frac{S_{\mathrm{bio}}^{\mathrm{int}}+S_{\mathrm{align}}}{2}. \end{array}$$

We assign equal weights to biological conservation and modality alignment because both are required for an integrated representation that aligns modalities while preserving cell identity. This choice gives neither component a predefined priority; component scores are also reported separately to make their contributions to the overall ranking explicit.

Cross-modal label transfer is evaluated using a five-nearest-neighbor classifier with cosine distance and uniform voting on $\ell_{2}$-normalized embeddings. Each modality serves alternately as the reference and query. Direction-specific accuracy and its bidirectional mean are calculated as

(25) $$\begin{array}{cc} A_{u\to v} & =\frac{1}{|Q_{v}|}\sum_{i\in Q_{v}}1\left({\hat{y}}_{i}^{u\to v}=y_{i}^{v}\right),\\ S_{\mathrm{transfer}} & =\frac{A_{r\to a}+A_{a\to r}}{2}, \end{array}$$

where *u* and *v* denote the reference and query modalities, respectively. The set $Q_{v}$ contains query cells whose annotated types occur in the reference modality, $y_{i}^{v}$ is the annotated type, and ${\hat{y}}_{i}^{u\to v}$ is the predicted type. Transfer accuracy is reported separately from the overall integration score.

#### 2.4.3. Translation Performance

Translation is evaluated at both the representation and molecular-profile levels. Translated representations refer to latent vectors mapped into the target modality space, whereas predicted molecular profiles refer to the corresponding decoder outputs. At the representation level, NMI, ARI, cASW, and cLISI are calculated separately on ${\mathrm{Map}}_{r\to a}\left(\mu^{r}\right)$ and ${\mathrm{Map}}_{a\to r}\left(\mu^{a}\right)$, using the corresponding source-cell annotations. Each evaluation uses only the translated embeddings of one direction, without combining them with observed target-cell embeddings. The translation biological conservation score is

(26) $$S_{\mathrm{bio}}^{u\to v}=\frac{1}{4}\sum_{q\in B}S_{q}^{u\to v},\;\left(u,v\right)\in \left\{\left(r,a\right),\left(a,r\right)\right\},$$

where B is the four-metric set defined in Equation (19), and the superscript u→v indicates evaluation on the corresponding mapped embeddings. This score is reported as the mean for each translation direction. Integration and translation biological benchmark scores are multiplied by 100 for presentation, whereas cell-level PCC values remain on their original unitless scale.

To assess the contribution of individual objectives, we perform ablations on PBMC-10k. For integration, we remove $L_{\mathrm{adv}}$, $L_{\mathrm{stru}}$, or $L_{\mathrm{anc}}$ individually, remove $L_{\mathrm{stru}}$ and $L_{\mathrm{anc}}$ jointly, or retain only $L_{\mathrm{rep}}$. For unpaired translation, we remove $L_{\mathrm{self}}$, $L_{\mathrm{map}}$, or $L_{\mathrm{con}}$, and additionally evaluate $L_{\mathrm{con}}$-only and $L_{\mathrm{map}}$-only variants. The complete ablation results are reported in Appendix A.

At the molecular-profile level, cell-level Pearson correlation coefficients (PCCs) measure agreement between decoded predictions and observed target profiles. Features are restricted to the intersection shared by the reference data and all compared methods, and profiles are aligned by cell and feature identifiers. The count layer is used when available; otherwise, the stored data matrix is used. Each non-negative matrix is log(1+x)-transformed, whereas matrices containing negative values are retained on their supplied scale.

Let $q_{i}$ and ${\hat{q}}_{i}$ denote the processed observed and predicted profiles of cell *i* over the common target features. Their centered profiles are $q_{i}^{\;c}=q_{i}-{\bar{q}}_{i}1$ and ${\hat{q}}_{i}^{\;c}={\hat{q}}_{i}-{\bar{\hat{q}}}_{i}1$, where the bars denote means across features, 1 is an all-ones vector, and the superscript *c* denotes centering. Cell-level PCC is calculated as

(27) $$\rho_{i}=\frac{{\left(q_{i}^{\;c}\right)}^{T}{\hat{q}}_{i}^{\;c}}{{∥q_{i}^{\;c}∥}_{2}{∥{\hat{q}}_{i}^{\;c}∥}_{2}+\epsilon },$$

where ${∥\cdot ∥}_{2}$ denotes the Euclidean norm and $\epsilon =10^{-12}$ provides numerical stability. Cells with a squared centered norm below ϵ in either profile are excluded from the PCC distributions. RNA and ATAC prediction are evaluated separately.

## 3. Results

### 3.1. scAICT Balances Biological Conservation and Modality Alignment

**Figure 2:**
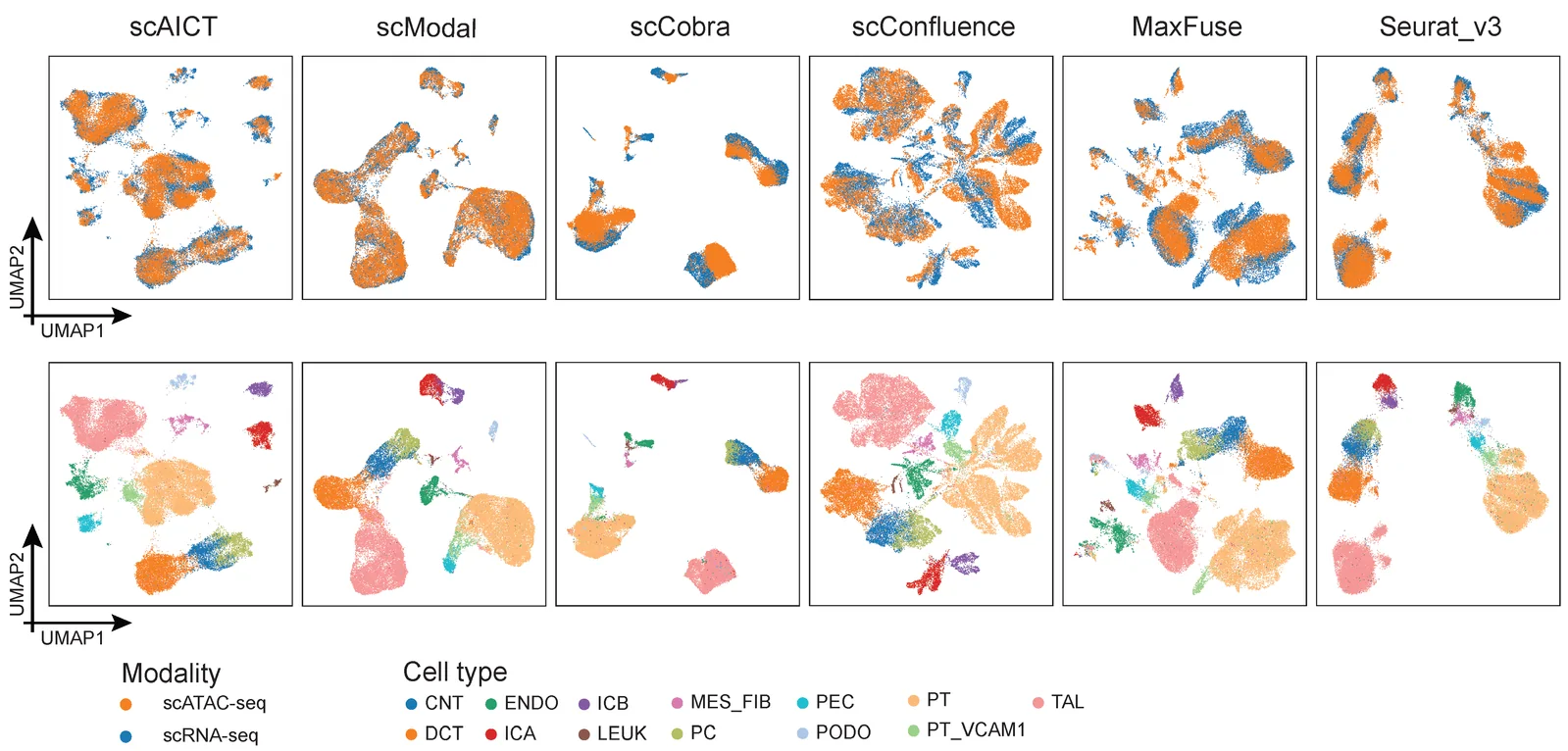
Joint embeddings of the Muto-2021 dataset generated by six integration methods. UMAPs are colored by modality (**top**) and cell type (**bottom**).

**Figure 3:**
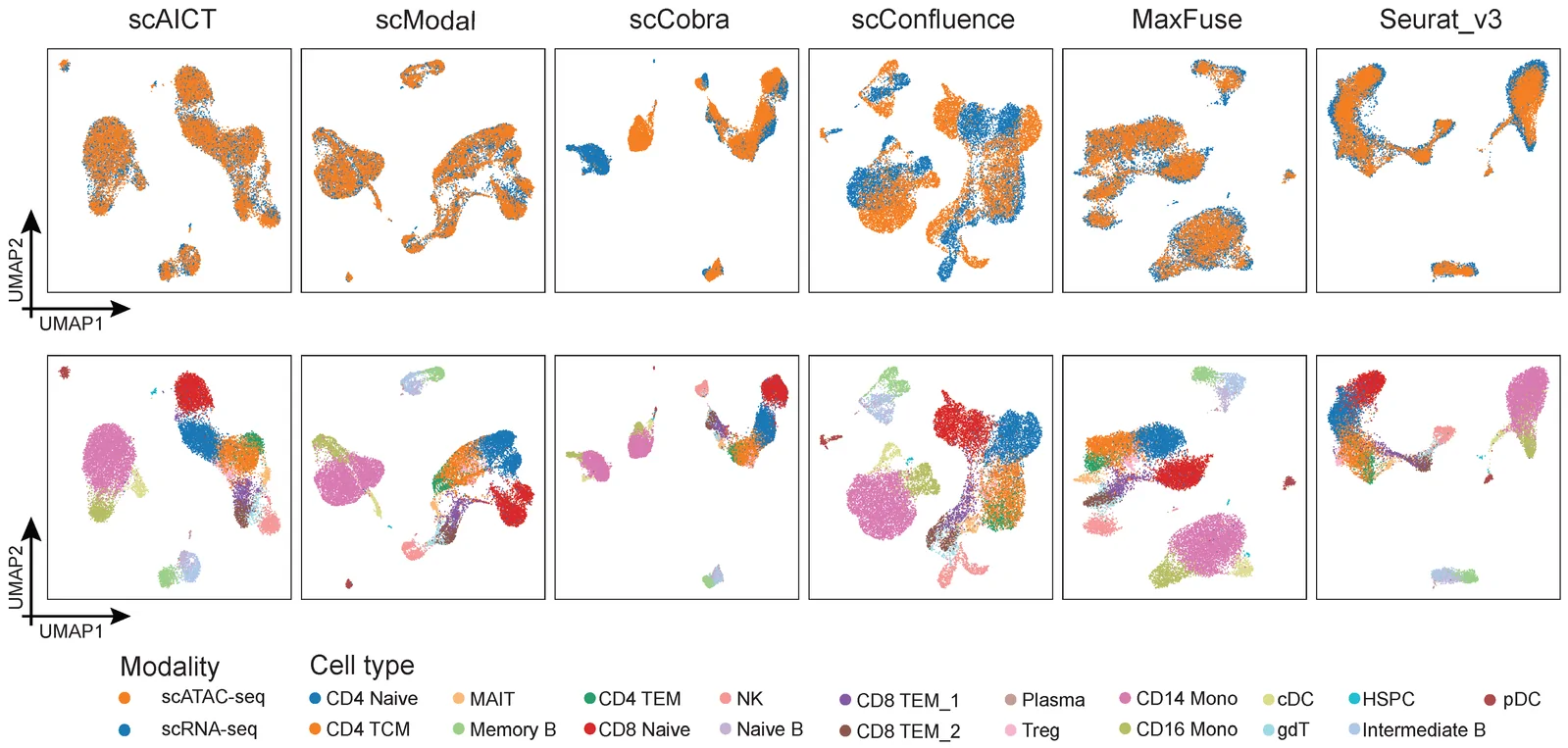
Joint embeddings of the PBMC-10k dataset generated by six integration methods. UMAPs are colored by modality (**top**) and cell type (**bottom**).

scAICT aligns independently measured gene expression and chromatin accessibility profiles while retaining recognizable cell populations in the naturally unpaired Muto-2021 kidney dataset (Figure 2). RNA and ATAC profiles occupy overlapping regions within the major thick ascending limb (TAL) and proximal tubule (PT) cell populations, while smaller populations such as podocytes (PODO) and parietal epithelial cells (PEC) remain distinguishable. Separation is less complete among some related populations, including connecting tubule (CNT) cells and principal cells (PC). Complementary integration of PBMC-10k, with native cell correspondences withheld, preserves major regions associated with monocytes, B cells, and T and natural killer cells (Figure 3). Related T-cell subsets occupy adjacent or continuous regions rather than uniformly isolated clusters. In the modality-colored panels, scAICT shows overlap across these major regions, whereas scCobra displays more visibly segregated modality-specific areas in both datasets. The UMAPs therefore suggest preservation of broad cell-type organization alongside modality mixing, which we quantify using biological conservation and alignment scores.

The quantitative results support the integration pattern observed in Muto-2021 dataset (Table 2). scAICT achieves the highest overall score of (78.62±0.17)%, compared with (76.88±0.32)% for scMODAL. This advantage reflects higher scores for both biological conservation, at (84.04±0.25)% versus (82.96±0.82)%, and modality alignment, at (73.21±0.39)% versus (70.79±1.09)%. NMI and ARI reach 87.29% and 89.69%, respectively, the highest values among the evaluated methods, supporting agreement with annotated kidney populations. scAICT also leads all four alignment metrics, with the largest margin over the respective runner-up in oASW, at 94.01% versus 88.72% for Seurat_v3.

The overall advantage is larger in PBMC-10k, where scAICT reaches (84.61±0.83)% compared with (79.51±0.53)% for scMODAL (Table 3). Here, modality alignment accounts for most of the difference, reaching (88.01±1.16)% versus (78.67±1.73)%. Gains of 16.18 and 10.92 percentage points over the respective runners-up in kBET and iLISI support improved local modality mixing. Biological conservation remains slightly higher than scMODAL, at (81.21±0.56)% versus (80.35±1.08)%, with the highest NMI, ARI, and cLISI values among the evaluated methods.

The main exception is cASW, for which scMODAL scores higher in both datasets, reaching 67.08% versus 60.48% in Muto-2021 and 62.32% versus 59.76% in PBMC-10k. Thus, scAICT’s highest aggregate scores reflect annotation agreement and modality mixing, despite lower silhouette-based cell-type separation than scMODAL.

Component ablations on PBMC-10k further show lower mean biological conservation and transfer accuracy after removal of individual integration objectives (Appendix A).

### 3.2. scAICT Improves Cross-Modal Cell-Type Label Transfer

**Figure 4:**
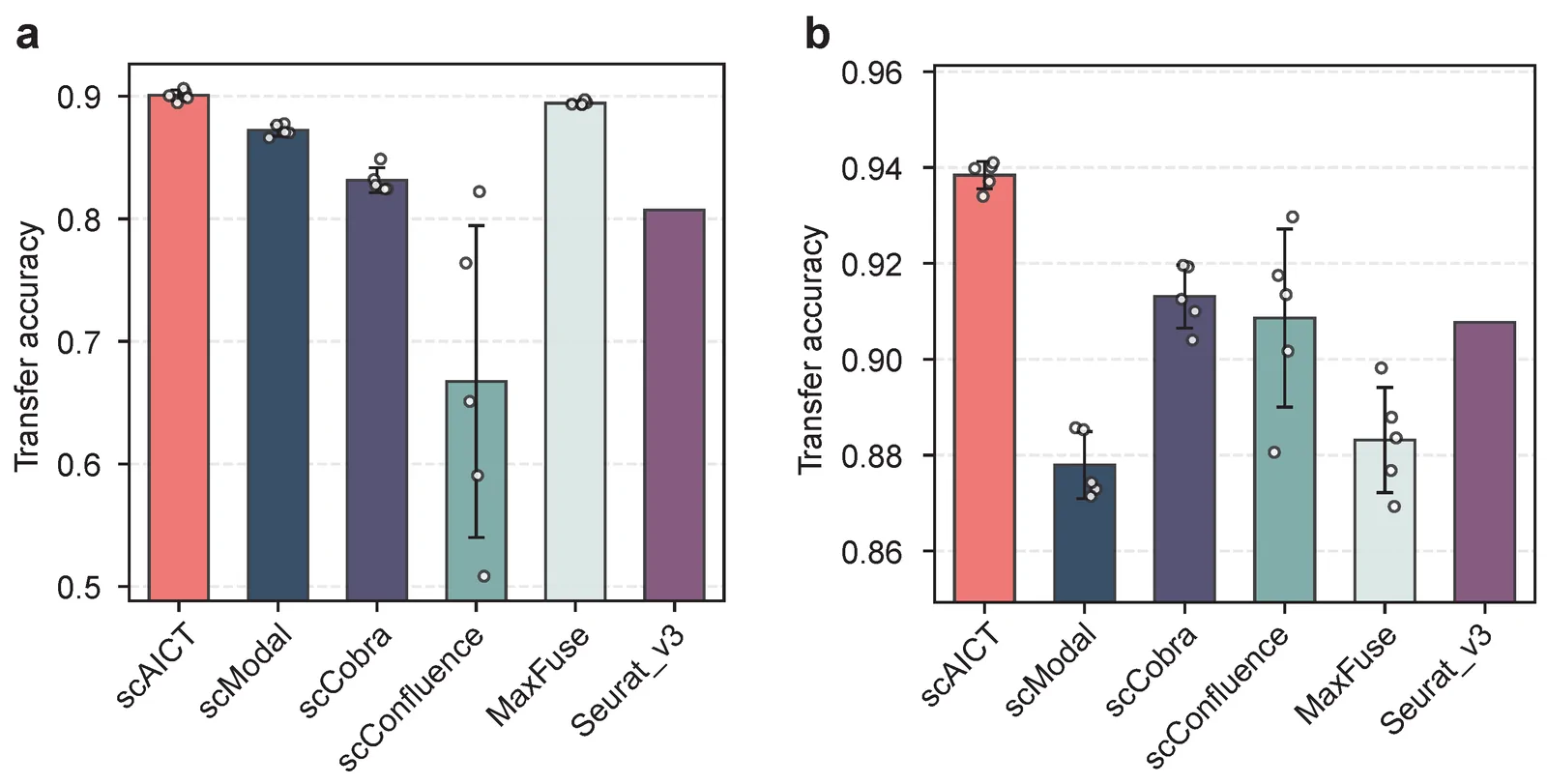
Bidirectional cross-modal cell-type label-transfer accuracy on (**a**) PBMC-10k and (**b**) Muto-2021. For each run, label-transfer accuracy is calculated separately using RNA profiles as the reference for ATAC annotation and ATAC profiles as the reference for RNA annotation, and the two accuracies are then averaged. Bars show the mean across five random seeds, error bars indicate standard deviations, and overlaid points represent individual runs. Seurat_v3 is evaluated once because its standard R-based workflow does not involve stochastic model training or random initialization, and is therefore shown without individual-run points or an error bar.

**Figure 5:**
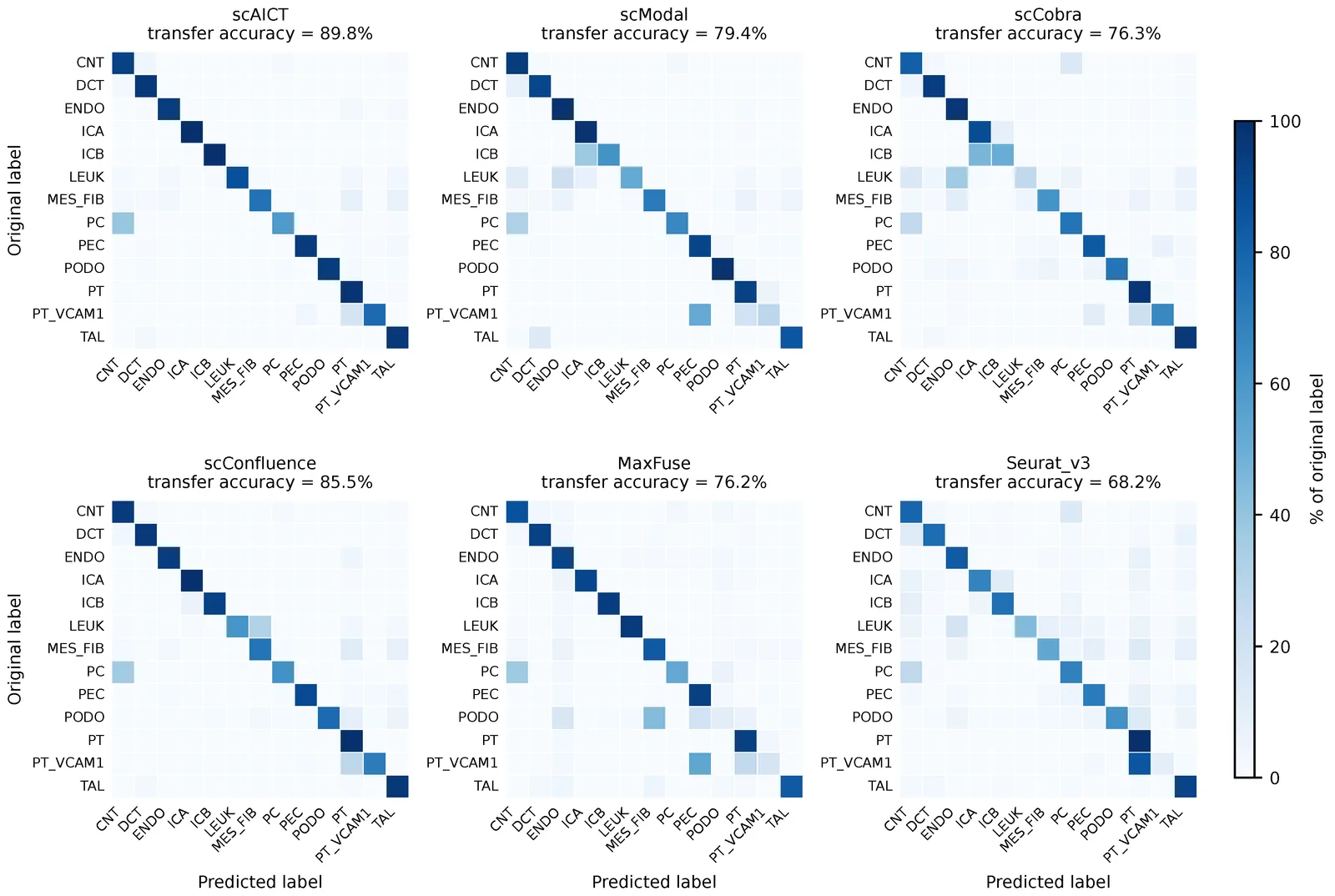
Cell-type-resolved RNA-guided annotation of ATAC profiles on Muto-2021 across six integration methods. Rows represent the ground-truth cell types of ATAC profiles, columns represent labels transferred from the RNA reference, and color indicates the percentage of cells within each ground-truth class. Label-transfer accuracy is reported above each matrix.

**Figure 6:**
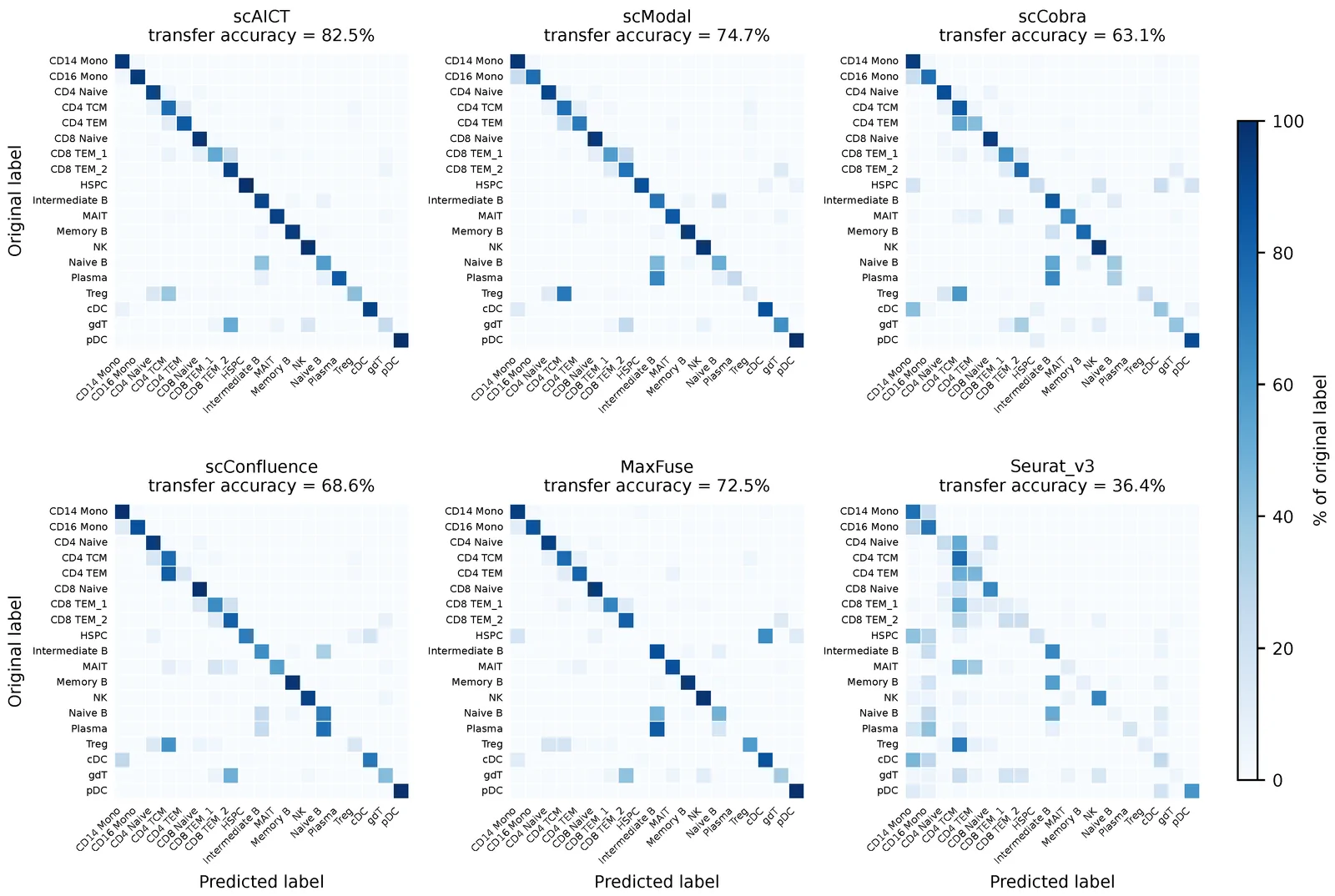
Cell-type-resolved RNA-guided annotation of ATAC profiles on PBMC-10k across six integration methods. Rows represent the ground-truth cell types of ATAC profiles, columns represent labels transferred from the RNA reference, and color indicates the percentage of cells within each ground-truth class. Label-transfer accuracy is reported above each matrix.

The integrated representations support cell-type annotation across modalities in both datasets. Cell-type labels are transferred from an annotated RNA reference to ATAC profiles and, in the reverse direction, from an annotated ATAC reference to RNA profiles. Mean bidirectional label-transfer accuracy summarizes performance across runs (Figure 4). Confusion matrices for RNA-guided annotation of ATAC profiles show cell-type-specific agreement and residual errors (Figure 5 and Figure 6).

Annotation transfer in Muto-2021 reaches approximately 94% mean bidirectional accuracy for scAICT, the highest among the evaluated methods, with limited variation across runs (Figure 4b). RNA-guided annotation of ATAC profiles achieves 89.8% accuracy in the confusion-matrix comparison, exceeding the next-best result from scConfluence by 4.3 percentage points (Figure 5). Most kidney populations retain strong diagonal signals, although PC and VCAM1-expressing proximal tubule cells (PT_VCAM1) remain sources of confusion. These results support the transfer of RNA-derived cell-type annotations to independently measured ATAC profiles.

Immune-cell annotation in PBMC-10k reaches approximately 90% mean bidirectional accuracy for scAICT, similar to MaxFuse (Figure 4a). When RNA profiles serve as the reference, scAICT assigns labels to ATAC profiles with 82.5% accuracy in the confusion-matrix comparison, compared with 74.7% for scMODAL and 72.5% for MaxFuse (Figure 6). Strong diagonal signals extend across most populations, while residual errors occur mainly among related T-cell states and B-cell subtypes. These results indicate that annotation transfer preserves broad cell identities across both datasets, although finer distinctions among related populations remain a source of error.

### 3.3. Unpaired Translation Preserves Cell-Type Structure and Molecular-Profile Concordance

Beyond annotation transfer, we evaluate whether scAICT preserves cell-type organization and molecular profiles during translation without native cell-level pairing or cell-type supervision. Monae serves as the baseline under these supervision conditions, predicting target profiles from shared cell representations. scAICT instead uses pseudo-pairs inferred during integration to supervise directional mapping networks before target-modality decoding, with separate integration and translation objectives. We evaluate these approaches on naturally unpaired Muto-2021 data and on PBMC-10k with native correspondences withheld. In this PBMC-10k evaluation, the RNA and ATAC profiles of the evaluated cells are present as separate observations during training, but their native correspondence is used only for evaluation. We first compare scAICT with Monae using the four biological conservation metrics calculated on translated representations (Table 4).

The Muto-2021 results show higher biological conservation for scAICT than Monae in both translation directions. RNA-to-ATAC scores reach (82.71±0.79)% and (80.92±0.93)%, respectively, whereas ATAC-to-RNA scores reach (81.64±0.91)% and (71.52±2.09)%. scAICT leads all four component metrics in both directions. The larger margin for ATAC-to-RNA indicates that its relative advantage is most pronounced in retaining cell-type organization when gene expression is predicted from chromatin accessibility. Because Muto-2021 lacks matched observations from individual cells, these results assess structural conservation rather than cell-level molecular accuracy.

With native PBMC-10k correspondences withheld, the same directional pattern appears. RNA-to-ATAC biological conservation scores are close, at (78.99±0.79)% for scAICT and (78.24±0.57)% for Monae. Monae has a slightly higher NMI, while scAICT has higher ARI, cASW, and cLISI. A larger separation occurs for ATAC-to-RNA, where scAICT reaches (78.05±0.65)% compared with (64.05±0.67)% for Monae and leads all four component metrics. The stronger relative performance in cell-type conservation for RNA prediction therefore extends across the naturally and simulated unpaired settings, while the smaller reverse-direction margins limit the scope of this advantage.

**Figure 7:**
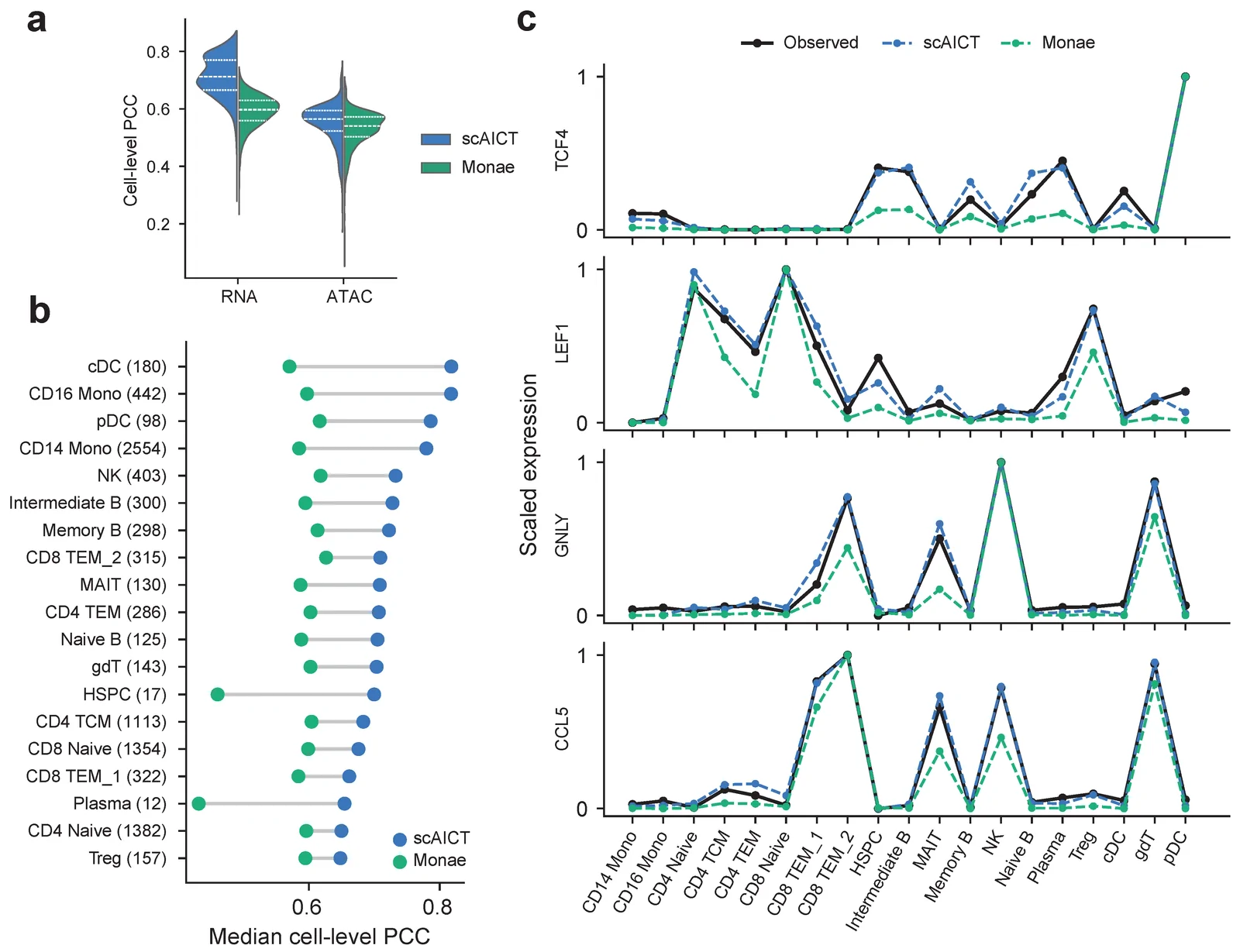
Molecular-profile concordance under unpaired training on PBMC-10k. Native cell correspondences are withheld during training and used only for evaluation. (**a**) Distributions of cell-level Pearson correlation coefficients (PCCs) between predicted and observed target profiles for RNA prediction from ATAC and ATAC prediction from RNA. Violin plots show results from a single run; white dashed lines indicate the 25th, 50th, and 75th percentiles. (**b**) Median cell-level PCC for RNA prediction within each annotated cell type. Numbers in parentheses indicate cell counts. (**c**) Scaled expression patterns of *TCF4*, *LEF1*, *GNLY*, and *CCL5* across cell types. Black solid lines denote observed RNA; blue and green dashed lines denote RNA predicted by scAICT and Monae, respectively.

We examine whether the advantage in cell-type conservation also extends to molecular-profile concordance. Using the native PBMC-10k matches as evaluation references, we calculate cell-level PCC between decoded predictions and observed target profiles. scAICT shows higher median PCC than Monae in both translation directions, with a larger separation for ATAC-to-RNA prediction and a smaller difference for RNA-to-ATAC prediction (Figure 7a). Thus, the comparative advantage extends beyond the organization of translated representations to agreement with observed molecular profiles.

For RNA prediction, scAICT achieves higher median cell-level PCC than Monae across all 19 annotated cell types (Figure 7b). Large differences are also evident in low-abundance populations, including plasma cells (12 cells) and hematopoietic stem and progenitor cells (HSPCs; 17 cells), indicating that the advantage is not restricted to well-represented cell types. Selected marker-gene expression patterns provide a complementary view of this agreement. These genes represent distinct immune-cell identities and states: *TCF4* is associated with plasmacytoid dendritic cells, *LEF1* with naive T cells, and *GNLY* and *CCL5* with cytotoxic lymphocytes, including NK cells and cytotoxic T cells. Across cell types, scAICT more closely follows the observed expression patterns of these genes than Monae, although deviations remain for individual populations (Figure 7c).

Complementary ablations on PBMC-10k show that the complete translation objective achieves the highest mean biological conservation and cell-level PCC in both directions among the tested variants (Appendix A). An evaluation of inferred pseudo-pairs further characterizes exact-cell matching and cell-type agreement across PBMC-10k populations (Appendix B).

### 3.4. Unpaired Translation Performance on Cells Held out from Translation Training

A separate evaluation assesses molecular prediction for cells excluded from translation training. After integration using all PBMC-10k cells, we randomly split cells into translation training and test sets at a 7:3 ratio, withholding both RNA and ATAC profiles of 2889 test cells from translation training. scAICT and Monae use the same cell split. Native cell correspondences are withheld during unpaired training and used only to align predicted and observed target profiles for evaluation.

**Figure 8:**
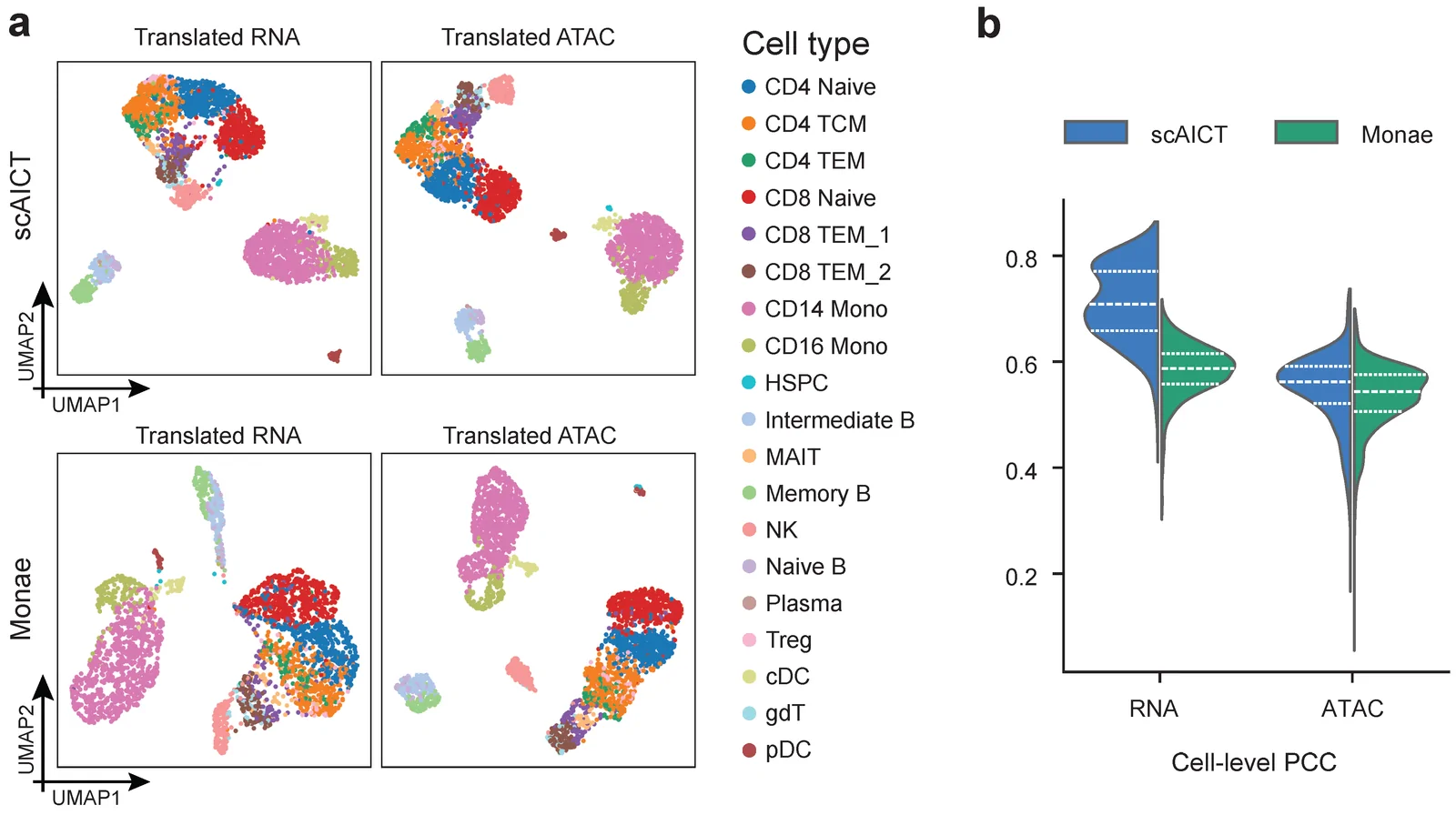
Cell-type organization and molecular-profile concordance in PBMC-10k cells held out from unpaired translation training. Native cell correspondences are used only for evaluation. (**a**) UMAPs of translated representations for ATAC-to-RNA prediction (**left**) and RNA-to-ATAC prediction (**right**), colored by cell type. Rows show scAICT (**top**) and Monae (**bottom**). (**b**) Cell-level PCC distributions between predicted and observed RNA or ATAC profiles for scAICT and Monae. Violin plots show results from a single run; white dashed lines indicate the 25th, 50th, and 75th percentiles.

scAICT retains cell-type organization in the translated representations of these cells (Figure 8a). For ATAC-to-RNA translation, its biological conservation score reaches (77.93±1.03)%, compared with (68.92±0.85)% for Monae, with higher means across all four component metrics (Table 5). For RNA-to-ATAC translation, the aggregate scores are closer, at (77.63±0.54)% and (76.76±0.65)%, respectively. scAICT achieves higher ARI and cASW, whereas Monae has slightly higher NMI and higher cLISI. The advantage in cell-type conservation therefore remains more pronounced for RNA prediction.

Molecular-profile evaluation further shows higher median cell-level PCC for scAICT than Monae in both translation directions, with a larger separation for RNA prediction and a smaller difference for ATAC prediction (Figure 8b). Together, these results support molecular prediction and preservation of cell-type structure in PBMC-10k cells excluded from translation training, following integration that includes these cells.

### 3.5. scAICT Preserves Cell-Type Structure in the Paired PBMC-10k Translation Benchmark

**Figure 9:**
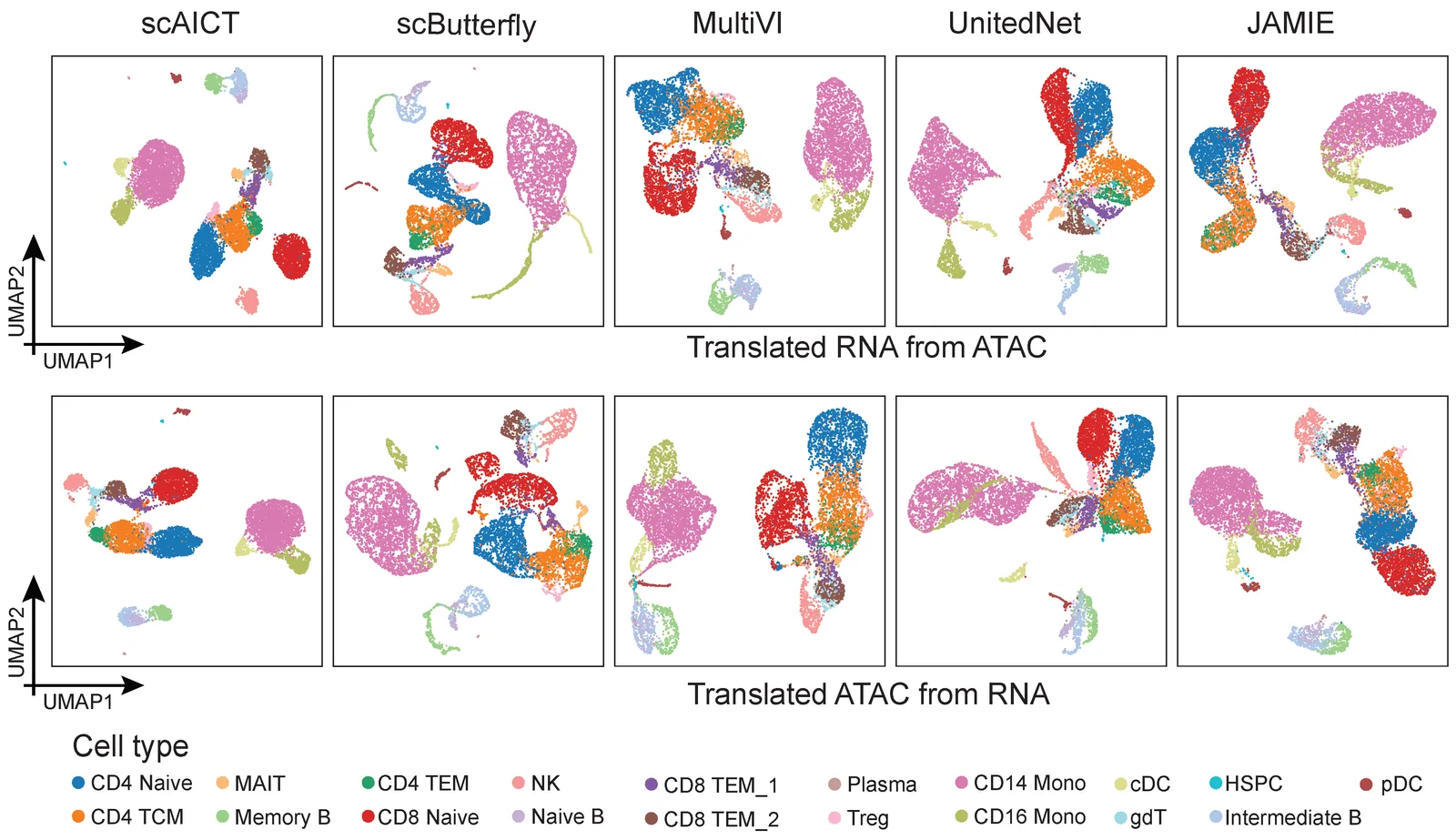
Cell-type organization of translated representations across five methods in the paired PBMC-10k benchmark. Native cell correspondences are available during translation training. UMAPs show representations for ATAC-to-RNA translation (**top**) and RNA-to-ATAC translation (**bottom**), colored by cell type.

To complement the unpaired evaluations, we benchmark scAICT against four translation methods in the paired PBMC-10k setting, where scAICT uses native cell correspondences for translation training. In both directions, scAICT retains distinguishable cell populations in the translated representations, while several competing methods show greater overlap or fragmentation (Figure 9). The following scores quantify cell-type structure in direction-specific translated representations before UMAP projection, using the corresponding source-cell annotations.

Cell-type organization remains well preserved when scAICT maps ATAC representations into the RNA latent space. The biological conservation score reaches (85.92±0.39)%, the highest among the evaluated methods, compared with (82.67±1.56)% for scButterfly (Table 6). NMI and ARI reach (89.88±0.30)% and (90.39±1.17)%, respectively, and cLISI reaches (97.74±0.04)%, all the highest means in this comparison. scButterfly retains a higher cASW.

This advantage in aggregate cell-type conservation also holds in the reverse direction. For RNA-to-ATAC translation, scAICT scores (84.54±0.72)%, compared with (80.66±1.55)% for scButterfly (Table 7). scAICT again leads NMI and ARI, reaching (87.43±1.05)% and (87.77±1.74)%, respectively. scButterfly leads cASW and cLISI, indicating that the methods differ in the structural properties they preserve most strongly. Across both directions, scAICT provides the highest aggregate score, with its clearest advantage in agreement between clustering and annotated cell types.

The scAICT biological conservation scores also exceed the observed-data references of 80.64% for RNA and 80.46% for ATAC, indicating clearer organization by annotated cell type in the evaluated representations. Molecular-profile agreement is examined separately below.

### 3.6. Paired Translation Retains Cell-Level Concordance and Cell-Type-Specific Molecular Patterns

**Figure 10:**
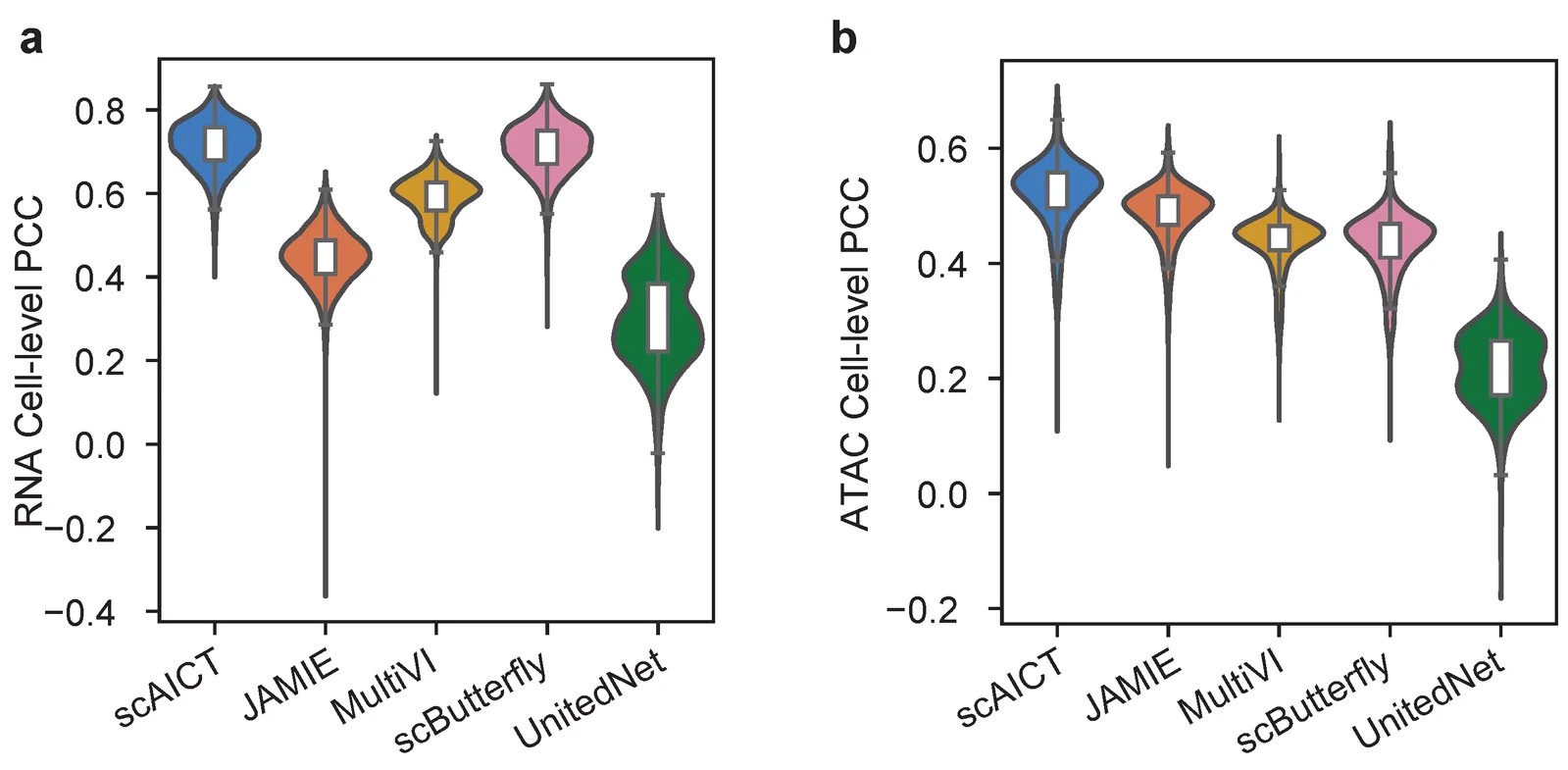
Cell-wise concordance between translated and original profiles in the paired PBMC-10k benchmark. Native cell correspondences are available during translation training. Violin plots show Pearson correlation coefficients for (**a**) RNA translated from ATAC and (**b**) ATAC translated from RNA.

The molecular-profile analyses extend the paired PBMC-10k benchmark to decoded predictions. With native cell correspondences used for translation training, scAICT retains cell-level agreement with the observed target profiles (Figure 10). PCC is calculated across common target features after predictions are aligned with the matched observations. For ATAC-to-RNA translation, scAICT shows high concordance comparable to the strongest competing method (Figure 10a). For RNA-to-ATAC translation, its PCC distribution shows the highest central tendency among the evaluated methods (Figure 10b). ATAC predictions generally have lower correlations than RNA predictions under this evaluation, indicating different levels of molecular concordance between the two target modalities.

**Figure 11:**
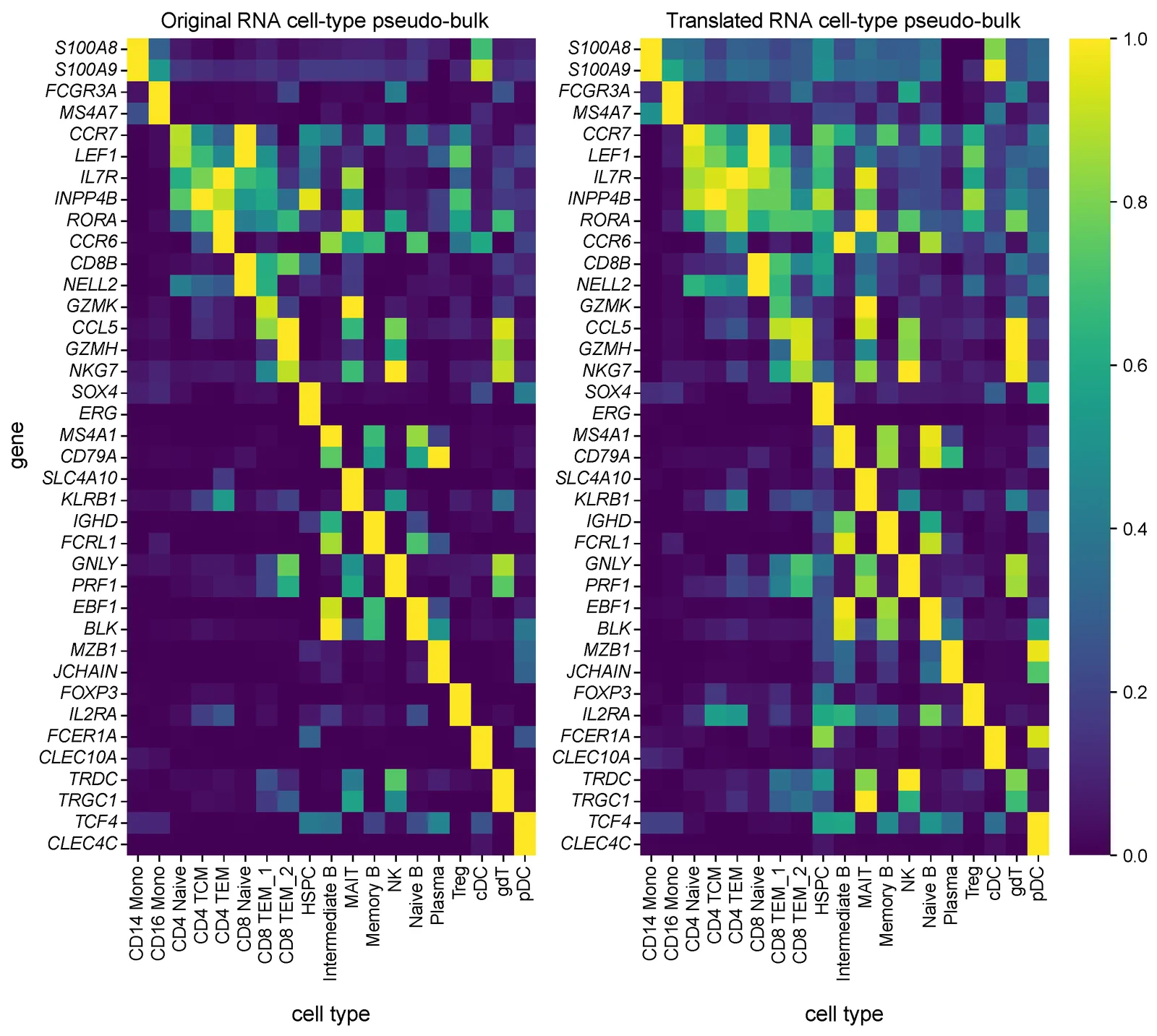
Cell-type pseudobulk marker-gene patterns in the original and scAICT-translated PBMC-10k RNA profiles following paired training. Heatmaps show the original RNA profiles (**left**) and RNA profiles translated from ATAC by scAICT (**right**). Expression values are scaled from 0 to 1.

Cell-type pseudobulk profiles also retain recognizable marker-gene patterns in RNA translated from ATAC (Figure 11). The translated profiles retain enrichment of *S100A8* and *S100A9* in CD14 monocytes and *FCGR3A* and *MS4A7* in CD16 monocytes. The B-cell-associated patterns of *MS4A1* and *CD79A* are also preserved. Markers of plasma cells, dendritic cells, regulatory T cells, and cytotoxic lymphocytes show corresponding enrichment in the translated profiles. These patterns connect molecular agreement with transcriptional differences among the annotated immune populations.

The translated heatmap nevertheless appears smoother than the observed heatmap, and some marker signals extend across related cell populations. Broad marker–cell-type associations remain recognizable, whereas finer expression contrasts are less completely recovered.

## 4. Discussion

scAICT connects integration and bidirectional translation through correspondence inferred from unpaired single-cell data. Integration supplies pseudo-pairs for an independently initialized translation model, allowing cross-modal relationships to guide prediction without transferring integration-stage parameters. The Muto-2021 results support preservation of cell-type structure, while PBMC-10k provides direct molecular evaluation under unpaired training alongside a complementary paired benchmark. Together, these findings support inferred correspondence as a practical source of supervision for molecular prediction within the evaluated settings.

The integration and annotation-transfer results highlight the importance of considering both modality alignment and biological conservation. Improved mixing is most useful when the shared representation also retains distinctions that support cell-type assignment. The ablations provide evidence for complementary contributions from the integration objectives (Appendix A). Adversarial alignment primarily supports modality mixing, whereas removing cross-modal anchors substantially reduces annotation-transfer accuracy and increases its variability. The structural constraint provides smaller improvements in biological conservation and transfer accuracy. During translation, self-reconstruction and contrastive matching support cell-type structure, while mapped-latent regularization contributes most clearly to molecular concordance. These different effects support combining objectives that constrain cellular organization and molecular prediction.

The unpaired translation results show a direction-dependent advantage over Monae. In PBMC-10k, the larger improvements for RNA prediction occur in both cell-type conservation and molecular-profile concordance, linking the structural comparison to agreement with observed measurements. The same directional pattern persists in cells excluded from translation training. These results complement Monae’s prediction from shared cell representations with evidence for pseudo-pair-supervised directional mapping, although the comparison does not isolate which architectural or training differences produce the advantage. The smaller RNA-to-ATAC margins also limit a general claim of superiority across prediction tasks.

The pseudo-pair analysis helps define the supervision supplied by integration (Appendix B). Cell-type agreement exceeds exact-cell matching across populations, and the true counterpart is recovered more frequently within the 50 nearest neighbors than as the single nearest neighbor. This pattern suggests that inferred pairs can support cell-type-associated molecular prediction without reliably recovering individual-cell identity. Lower agreement in several T-cell populations identifies a potential source of imprecise supervision.

Marker-expression analyses under unpaired and paired training connect molecular concordance with recognizable immune-cell patterns. However, the paired-training heatmap shows smoother expression and some extension of marker signals across related populations, indicating incomplete recovery of finer contrasts. Thus, preservation of cell-type-associated information does not establish accurate recovery of every molecular feature or individual-cell difference.

Several limitations define the scope of these findings. The absence of matched measurements in Muto-2021 limits its translation evaluation to cell-type conservation, with direct molecular validation confined to PBMC-10k. Molecular evaluation also focuses on the selected genes and associated accessible regions, and does not establish prediction accuracy across the full transcriptome or accessibility landscape. The held-out analysis assesses prediction for cells excluded from translation training following integration of the full dataset. More broadly, the two datasets represent a limited range of tissues and cellular compositions. Evaluation across additional tissues, cell compositions, and experimental settings would help establish the broader applicability of scAICT.

## 5. Conclusions

scAICT uses correspondence inferred during integration to guide bidirectional translation of unpaired single-cell gene expression and chromatin accessibility profiles. Evaluations on Muto-2021 and PBMC-10k support integration, annotation transfer, and preservation of cell-type structure. Under unpaired training in PBMC-10k, scAICT achieves higher mean biological conservation and higher median cell-level molecular correlations than Monae in both directions, including in cells excluded from translation training, with larger advantages for RNA prediction. Inferred pseudo-pairs preserve cell-type correspondence more consistently than exact cell identity, defining both their potential value as supervision and their resolution limits. These findings support prediction of cell-type-associated molecular profiles from unpaired data, with direct molecular validation confined to PBMC-10k and kidney evidence limited to structural conservation.

## Figures and Tables

**Table 1:** Details of the datasets used in this study. Input feature counts refer to the annotated matrices before preprocessing in this study. Cell-type counts are reported per modality.

Dataset	RNA Profiles	ATAC Profiles	Cell Types	Input Genes	Input Peaks	Selected Genes	Selected Peaks
Muto-2021	19,985	24,205	13	27,146	99,019	3000	18,229
PBMC-10k	9631	9631	19	29,095	107,194	3000	17,873

**Table 2:** Benchmark comparison of integration performance on Muto-2021. Values show the mean ± standard deviation across five random seeds, except for Seurat_v3, which is evaluated once; n.a. indicates that the standard deviation is not available. The highest mean in each metric is shown in bold.

Method	NMI (%)	ARI (%)	cASW (%)	cLISI (%)	Biological Conservation (%)	kBET (%)	GC (%)	oASW (%)	iLISI (%)	Modality Alignment (%)	Overall Score (%)
scAICT	87.29±0.52	89.69±0.97	60.48±0.11	98.68±0.04	84.04±0.25	45.03±0.85	98.77±0.07	94.01±0.24	55.04±0.78	73.21±0.39	78.62±0.17
scMODAL	81.48±0.86	85.00±2.48	67.08±0.13	98.29±0.03	82.96±0.82	44.33±2.30	98.22±0.16	87.11±0.66	53.51±1.81	70.79±1.09	76.88±0.32
scCobra	83.73±0.41	87.84±0.66	59.68±0.34	97.65±0.14	82.23±0.31	13.43±1.78	97.85±0.15	80.54±0.91	21.27±2.11	53.27±1.09	67.75±0.49
scConfluence	81.93±0.29	79.16±2.71	64.36±1.11	98.47±0.13	80.98±0.45	11.77±2.54	96.79±1.08	82.72±1.59	16.10±3.56	51.84±1.68	66.41±0.77
MaxFuse	79.63±2.05	84.66±2.96	60.11±0.35	98.40±0.04	80.70±1.21	16.91±0.17	97.99±0.67	74.01±0.45	21.87±0.29	52.69±0.28	66.70±0.59
Seurat_v3	77.84±n.a.	82.92±n.a.	53.22±n.a.	95.57±n.a.	77.39±n.a.	31.03±n.a.	96.05±n.a.	88.72±n.a.	43.51±n.a.	64.83±n.a.	71.11±n.a.

**Table 3:** Benchmark comparison of integration performance on PBMC-10k. Values show the mean ± standard deviation across five random seeds, except for Seurat_v3, which is evaluated once; n.a. indicates that the standard deviation is not available. The highest mean in each metric is shown in bold.

Method	NMI (%)	ARI (%)	cASW (%)	cLISI (%)	Biological Conservation (%)	kBET (%)	GC (%)	oASW (%)	iLISI (%)	Modality Alignment (%)	Overall Score (%)
scAICT	84.04±0.70	83.59±1.01	59.76±0.61	97.45±0.17	81.21±0.56	79.55±2.68	99.20±0.14	94.36±0.56	78.94±1.57	88.01±1.16	84.61±0.83
scMODAL	81.33±1.67	80.46±2.62	62.32±0.31	97.29±0.16	80.35±1.08	63.37±3.92	97.62±1.15	85.68±0.50	68.02±2.96	78.67±1.73	79.51±0.53
scCobra	76.62±0.41	63.79±0.42	56.51±0.28	96.20±0.15	73.28±0.25	20.72±1.76	97.09±1.46	80.52±0.96	29.32±1.93	56.91±0.96	65.10±0.42
scConfluence	69.69±9.51	56.52±19.63	55.56±4.33	95.42±1.50	69.30±8.70	12.00±10.51	93.38±1.82	75.73±4.21	15.04±11.94	49.04±6.82	59.17±7.74
MaxFuse	82.74±0.36	82.90±0.73	57.63±0.34	96.77±0.08	80.01±0.22	31.42±0.47	94.54±1.39	83.81±0.57	40.83±0.37	62.65±0.45	71.33±0.15
Seurat_v3	75.18±n.a.	73.58±n.a.	55.23±n.a.	95.96±n.a.	74.99±n.a.	35.15±n.a.	95.08±n.a.	90.86±n.a.	46.13±n.a.	66.81±n.a.	70.90±n.a.

**Table 4:** Cell-type conservation in translated representations under unpaired training on PBMC-10k and Muto-2021. Native PBMC-10k correspondences are withheld during training. Values show the mean ± standard deviation across five random seeds. Within each dataset and translation direction, the higher mean between scAICT and Monae is shown in bold.

Dataset	Direction	Method	NMI (%)	ARI (%)	cASW (%)	cLISI (%)	Mean (%)
PBMC-10k	RNA→ATAC	scAICT	80.13±0.94	77.85±2.25	60.88±0.17	97.09±0.05	78.99±0.79
		Monae	80.65±0.60	76.19±2.34	59.64±0.23	96.49±0.05	78.24±0.57
	ATAC→RNA	scAICT	79.23±0.85	78.52±1.80	58.70±0.10	95.74±0.06	78.05±0.65
		Monae	63.83±0.49	43.93±2.16	54.73±0.25	93.73±0.27	64.05±0.67
Muto-2021	RNA→ATAC	scAICT	86.25±1.12	84.45±2.00	61.29±0.09	98.84±0.02	82.71±0.79
		Monae	82.55±1.19	83.69±1.94	60.23±0.57	97.20±0.17	80.92±0.93
	ATAC→RNA	scAICT	82.32±1.96	87.15±1.74	59.17±0.08	97.92±0.02	81.64±0.91
		Monae	67.96±1.42	66.42±7.25	56.89±0.53	94.79±0.47	71.52±2.09

**Table 5:** Cell-type conservation in translated representations of PBMC-10k cells held out from unpaired translation training. Values show the mean ± standard deviation across five random seeds. Within each translation direction, the higher mean between scAICT and Monae is shown in bold.

Direction	Method	NMI (%)	ARI (%)	cASW (%)	cLISI (%)	Mean (%)
RNA→ATAC	scAICT	78.95±1.06	76.14±1.41	60.31±0.62	95.10±0.19	77.63±0.54
	Monae	78.96±0.91	72.16±2.05	59.81±0.72	96.12±0.10	76.76±0.65
ATAC→RNA	scAICT	78.87±1.34	77.60±2.23	60.21±0.52	95.05±0.29	77.93±1.03
	Monae	70.84±1.19	53.05±2.86	58.03±0.47	93.78±0.15	68.92±0.85

**Table 6:** Cell-type structure in translated representations for ATAC-to-RNA translation in the paired PBMC-10k benchmark. Native cell correspondences are available during translation training. Scores are expressed as percentages and reported as mean ± standard deviation across five random seeds. Observed-data references are evaluated once; n.a. indicates that the standard deviation is not available. The Mean column denotes the biological conservation score. The highest mean in each metric is shown in bold.

Method	NMI	ARI	cASW	cLISI	Mean
Observed	83.10±n.a.	83.13±n.a.	59.94±n.a.	96.40±n.a.	80.64±n.a.
scAICT	89.88±0.30	90.39±1.17	65.68±0.29	97.74±0.04	85.92±0.39
scButterfly	82.43±1.59	80.49±4.40	70.50±0.69	97.27±0.20	82.67±1.56
UnitedNet	80.78±5.03	82.53±5.26	62.09±1.00	96.89±0.51	80.57±2.79
MultiVI	78.77±1.78	78.30±2.59	59.92±1.04	96.20±0.30	78.30±1.20
JAMIE	76.17±1.54	71.94±5.22	61.76±0.68	95.55±0.26	76.35±1.59

**Table 7:** Cell-type structure in translated representations for RNA-to-ATAC translation in the paired PBMC-10k benchmark. Native cell correspondences are available during translation training. Scores are expressed as percentages and reported as mean ± standard deviation across five random seeds. Observed-data references are evaluated once; n.a. indicates that the standard deviation is not available. The Mean column denotes the biological conservation score. The highest mean in each metric is shown in bold.

Method	NMI	ARI	cASW	cLISI	Mean
Observed	83.72±n.a.	83.70±n.a.	58.13±n.a.	96.28±n.a.	80.46±n.a.
scAICT	87.43±1.05	87.77±1.74	65.34±0.23	97.62±0.08	84.54±0.72
scButterfly	81.99±1.53	68.92±4.52	73.49±0.67	98.24±0.12	80.66±1.55
UnitedNet	79.30±1.78	70.97±5.93	61.40±2.55	97.63±0.48	77.32±2.18
MultiVI	78.80±1.22	78.87±1.33	61.16±0.08	96.19±0.25	78.75±0.66
JAMIE	78.74±0.97	73.61±4.13	60.79±0.99	96.34±0.06	77.37±1.34

**Figure A1:**
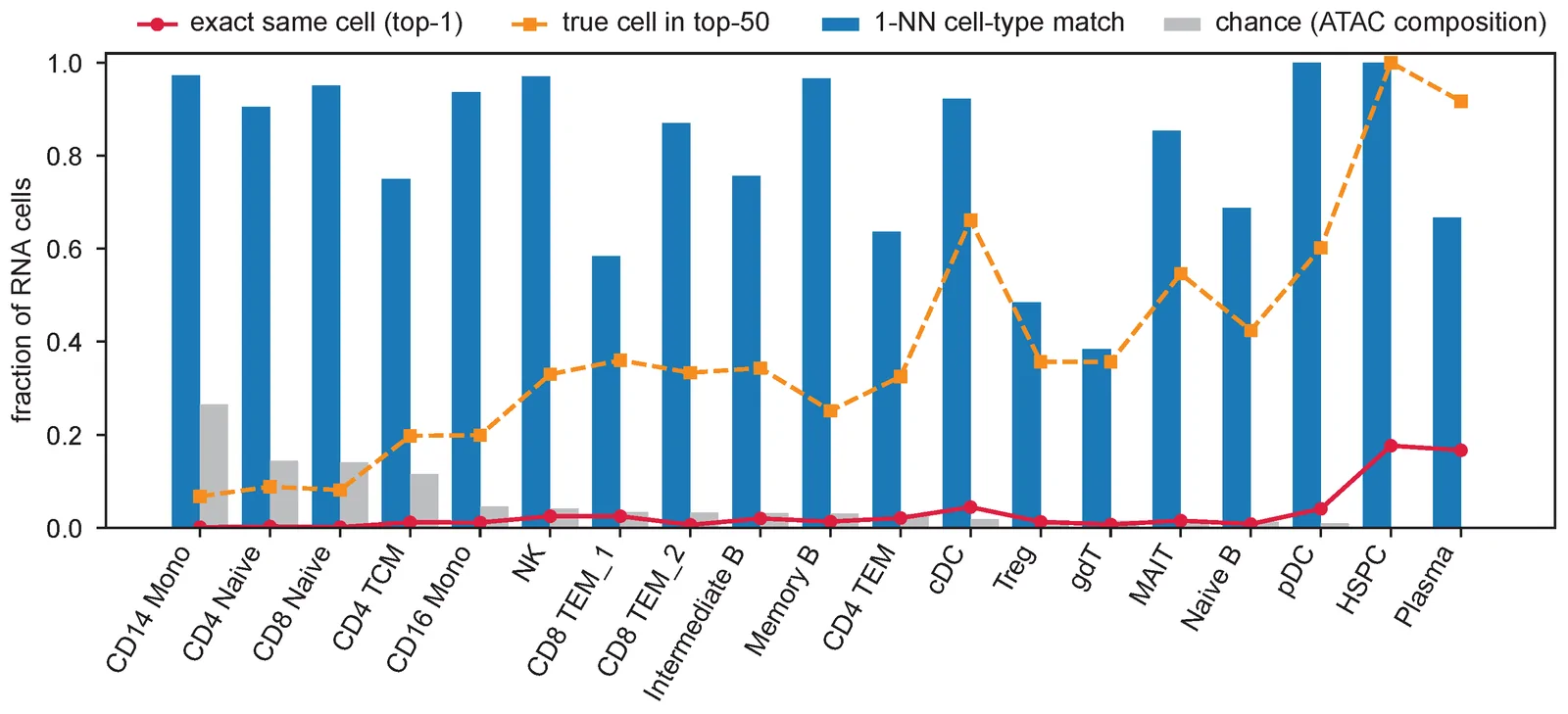
Pseudo-pair accuracy across PBMC-10k cell types. Blue bars show the fraction of RNA cells whose nearest ATAC neighbor has the same cell-type label; gray bars show the corresponding cell-type frequency among ATAC cells as a random-matching reference. Red circles show exact-match rates, and orange squares connected by a dashed line show the fraction of RNA cells whose true partner ranks among the 50 nearest ATAC cells. Cell types are ordered by decreasing RNA cell count.

## Data Availability

The preprocessed RNA and ATAC datasets analyzed in this study are obtained from the scGLUE data repository (https://scglue.readthedocs.io/en/latest/data.html, accessed on 6 October 2026), under the dataset names Muto-2021 and 10x-Multiome-Pbmc10k. Each dataset is provided as separate RNA and ATAC files in H5AD format. The original Muto-2021 sequencing data are available through GEO accession GSE151302 (https://www.ncbi.nlm.nih.gov/geo/query/acc.cgi?acc=GSE151302, accessed on 6 October 2026), with previously generated RNA profiles for three kidney samples deposited under GSE131882 (https://www.ncbi.nlm.nih.gov/geo/query/acc.cgi?acc=GSE131882, accessed on 6 October 2026). The original PBMC-10k data are available from 10x Genomics as PBMC from a Healthy Donor-Granulocytes Removed Through Cell Sorting (10k) (https://www.10xgenomics.com/datasets/pbmc-from-a-healthy-donor-granulocytes-removed-through-cell-sorting-10-k-1-standard-1-0-0, accessed on 6 October 2026) (Cell Ranger ARC 1.0.0).

## Appendix A. Ablation Studies

We evaluate integration and unpaired translation ablations on PBMC-10k using the same fixed set of five random seeds for the complete model and all ablation variants. Sharing the seed set supports controlled comparisons of the training objectives. This set differs from the five random seeds used in the main-text benchmarks, leading to differences in the reported full-model means and standard deviations. Variant names follow the objectives defined in Methods, and each removal is compared with the complete objective evaluated using the same seed set within the corresponding ablation experiment.

### Appendix A.1. Integration Objectives Affect Alignment and Label Transfer

Table A1 compares the complete integration objective, $L_{\mathrm{int}}$, with component removals and the representation-only objective, $L_{\mathrm{rep}}$.

**Table A1:** Ablation study of multi-omics integration on PBMC-10k. Values are percentages and are reported as mean ± standard deviation across five random seeds; higher values indicate better performance, and the highest mean in each column is shown in bold.

Variant	Biological Conservation (%)	Modality Alignment (%)	Overall Score (%)	Transfer Accuracy (%)
$L_{\mathrm{int}}$	83.34±0.38	83.94±3.73	83.64±1.79	89.50±0.46
w/o $L_{\mathrm{adv}}$	78.31±0.96	44.06±1.92	61.19±1.39	77.24±5.26
w/o $L_{\mathrm{stru}}$	82.49±1.21	84.13±4.35	83.31±2.62	88.73±0.80
w/o $L_{\mathrm{anc}}$	73.16±9.65	77.12±11.41	75.14±10.37	51.80±35.17
w/o $\left(L_{\mathrm{stru}}+L_{\mathrm{anc}}\right)$	75.98±7.49	79.84±8.40	77.91±7.78	62.98±30.13
$L_{\mathrm{rep}}$ only	73.15±0.34	24.08±1.13	48.61±0.65	11.54±8.21

The largest integration changes distinguish the roles of adversarial alignment and cross-modal anchors. Removing $L_{\mathrm{adv}}$ reduces modality alignment by 39.88 percentage points and transfer accuracy by 12.26 percentage points, highlighting its role in cross-modal alignment. Removing $L_{\mathrm{anc}}$ decreases transfer accuracy from (89.50±0.46)% to (51.80±35.17)% and lowers biological conservation by 10.18 percentage points. The lower mean and greater variability support the contribution of anchors to accurate and consistent cell-type correspondence.

The structural term provides a smaller benefit to biological conservation and transfer accuracy, whose means decrease by 0.85 and 0.77 percentage points upon removal. Modality alignment increases slightly without this term, indicating that its contribution is specific to preserving biological structure rather than improving every metric. Both joint structural–anchor removal and representation-only learning yield lower overall and transfer scores than the complete objective. Joint removal performs better than anchor removal alone, with substantial variability in both variants, so these effects are not simply additive. Overall, the complete integration objective provides the highest mean biological conservation, overall score, and transfer accuracy among the tested variants.

### Appendix A.2. Translation Objectives Support Structure and Molecular Concordance

Table A2 compares the complete translation objective, $L_{\mathrm{trans}}$, with component removals and single-objective variants.

**Table A2:** Ablation study of cross-modal translation on PBMC-10k under the unpaired setting. Biological conservation is evaluated on translated representations and reported as percentages; cell-level PCC is evaluated on decoded molecular profiles and reported on its original unitless scale. Values are reported as mean ± standard deviation across five random seeds; higher values indicate better performance, and the highest mean in each column is shown in bold.

	RNA → ATAC		ATAC → RNA	
Variant	Biological Conservation (%)	Cell-Level PCC	Biological Conservation (%)	Cell-Level PCC
$L_{\mathrm{trans}}$	81.17±0.99	0.6049±0.0028	80.44±0.50	0.8301±0.0017
w/o $L_{\mathrm{self}}$	79.45±1.06	0.6039±0.0023	79.95±0.77	0.8289±0.0010
w/o $L_{\mathrm{map}}$	80.58±0.85	0.5598±0.0026	79.36±0.32	0.8207±0.0020
w/o $L_{\mathrm{con}}$	80.93±0.65	0.6003±0.0024	78.83±0.79	0.8277±0.0016
$L_{\mathrm{con}}$ only	79.19±0.63	0.5304±0.0032	77.96±1.09	0.7315±0.0060
$L_{\mathrm{map}}$ only	80.63±0.71	0.5761±0.0028	78.14±0.93	0.7712±0.0060

Preservation of cell-type structure depends on different translation objectives in the two directions. Removing $L_{\mathrm{self}}$ produces the largest decrease in RNA-to-ATAC biological conservation among individual removals, at 1.72 percentage points. Removing $L_{\mathrm{con}}$ produces the largest corresponding decrease for ATAC-to-RNA, at 1.61 percentage points. These directional effects support the contribution of both objectives to preserving cell-type structure.

Mapped-latent regularization contributes most clearly to molecular concordance. Removing $L_{\mathrm{map}}$ reduces PCC from 0.6049±0.0028 to 0.5598±0.0026 for RNA-to-ATAC and from 0.8301±0.0017 to 0.8207±0.0020 for ATAC-to-RNA. Both decreases are the largest among individual component removals. The complete objective also exceeds both single-objective variants in all four reported means, supporting their combined use for structural preservation and molecular prediction.

## Appendix B. Pseudo-Pair Accuracy

We evaluate inferred pseudo-pairs on PBMC-10k using native cell correspondences withheld during integration. Each RNA cell is assigned its nearest ATAC neighbor by cosine distance in the shared latent space, as described in Section 2.1.3. For each cell type, we assess exact-match accuracy, cell-type agreement, and the fraction of RNA cells whose true ATAC partner falls within the 50 nearest neighbors (Figure A1).

Cell-type agreement exceeds exact-match accuracy and the cell-type-specific random-matching reference across all populations. Agreement is highest for pDCs and HSPCs and remains high for CD14 and CD16 monocytes, whereas several T-cell populations, particularly Tregs and gdT cells, show lower agreement. Exact-match rates are low, but the true partner is recovered more frequently within the 50 nearest ATAC neighbors, most prominently for HSPCs and plasma cells. This candidate set comprises approximately 0.5% of the 9631 ATAC cells, indicating that the true counterpart can lie within a small local neighborhood even when it is not the nearest neighbor. The extent of this recovery varies across populations. Together with the higher cell-type agreement, this pattern suggests that pseudo-pairs can provide supervision for cell-type-associated molecular features without reliably recovering individual-cell identity. It does not establish equivalence between the inferred and true target profiles.
